# Inspection of Floating Offshore Wind Turbines Using Multi-Rotor Unmanned Aerial Vehicles: Literature Review and Trends

**DOI:** 10.3390/s24030911

**Published:** 2024-01-30

**Authors:** Kong Zhang, Vikram Pakrashi, Jimmy Murphy, Guangbo Hao

**Affiliations:** 1 School of Engineering and Architecture, University College Cork, T12 K8AF Cork, Ireland; kong.zhang@umail.ucc.ie (K.Z.); jimmy.murphy@ucc.ie (J.M.); 2 Marine and Renewable Energy Ireland, Environmental Research Institute, University College Cork, P43 C573 Cork, Ireland; 3 UCD Centre for Mechanics, Dynamical Systems and Risk Laboratory, School of Mechanical and Materials Engineering, University College Dublin, D04 V1W8 Dublin, Ireland; vikram.pakrashi@ucd.ie

**Keywords:** UAV, floating offshore wind turbine, O&M, CM, robotics, aerial manipulator

## Abstract

Operations and maintenance (O&M) of floating offshore wind turbines (FOWTs) require regular inspection activities to predict, detect, and troubleshoot faults at high altitudes and in harsh environments such as strong winds, waves, and tides. Their costs typically account for more than 30% of the lifetime cost due to high labor costs and long downtime. Different inspection methods, including manual inspection, permanent sensors, climbing robots, remotely operated vehicles (ROVs), and unmanned aerial vehicles (UAVs), can be employed to fulfill O&M missions. The UAVs, as an enabling technology, can deal with time and space constraints easily and complete tasks in a cost-effective and efficient manner, which have been widely used in different industries in recent years. This study provides valuable insights into the existing applications of UAVs in FOWT inspection, highlighting their potential to reduce the inspection cost and thereby reduce the cost of energy production. The article introduces the rationale for applying UAVs to FOWT inspection and examines the current technical status, research gaps, and future directions in this field by conducting a comprehensive literature review over the past 10 years. This paper will also include a review of UAVs’ applications in other infrastructure inspections, such as onshore wind turbines, bridges, power lines, solar power plants, and offshore oil and gas fields, since FOWTs are still in the early stages of development. Finally, the trends of UAV technology and its application in FOWTs inspection are discussed, leading to our future research direction.

## 1. Introduction

### 1.1. Background of FOWT

**Figure 1:**
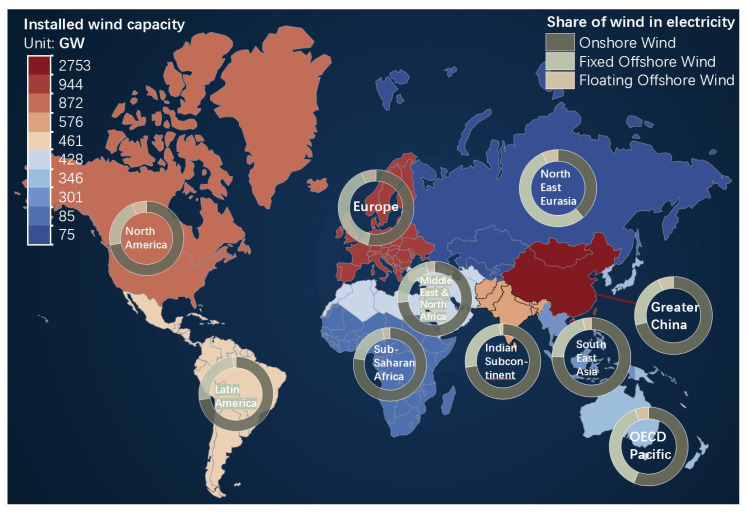
The installed wind capacity and the share of wind in electricity generation in 2050 vary by region. (Original figure. Data from Det Norske Veritas [2] and the Global Wind Energy Council [3]).

Due to global warming, the energy crisis, and geopolitical factors, wind energy is becoming one of the most vital and accessible sustainable and renewable energy sources for the foreseeable future [1]. As of 2022, wind power accounted for 6% of the world’s electricity production, almost exclusively in the form of onshore wind, according to Det Norske Veritas [2], the world’s largest classification society, and the Global Wind Energy Council [3]. The global grid-connected wind capacity is projected to rise from 1600 TWh per year in 2020 to 19,000 TWh per year by 2050, supplying nearly 50% of all grid-connected electricity in Europe and 40% in North America, Latin America, and Greater China. However, finding suitable land for wind farms will increasingly pose challenges [4]. Offshore wind power has shown promise in recent years as it mitigates conflicts with human activity, social acceptance, visual impact, land use, environmental protection, and bird habitat [5,6]. The share of offshore wind of total global wind in the global wind power capacity is expected to grow from 8% in 2020 to 34% in 2050, reaching 2003 GW of installed capacity, including 300 GW of floating capacity [2]. Geographically, Greater China and Europe are projected to have the largest installed capacity of offshore wind by 2050, as shown in Figure 1. The Pacific countries and Europe have a larger proportion of offshore wind due to their high population density and abundant marine resources. These factors indicate a greater focus on research in these regions. In summary, wind power, particularly floating offshore wind power, will assume an increasingly significant role in the future energy mix.

**Figure 2:**
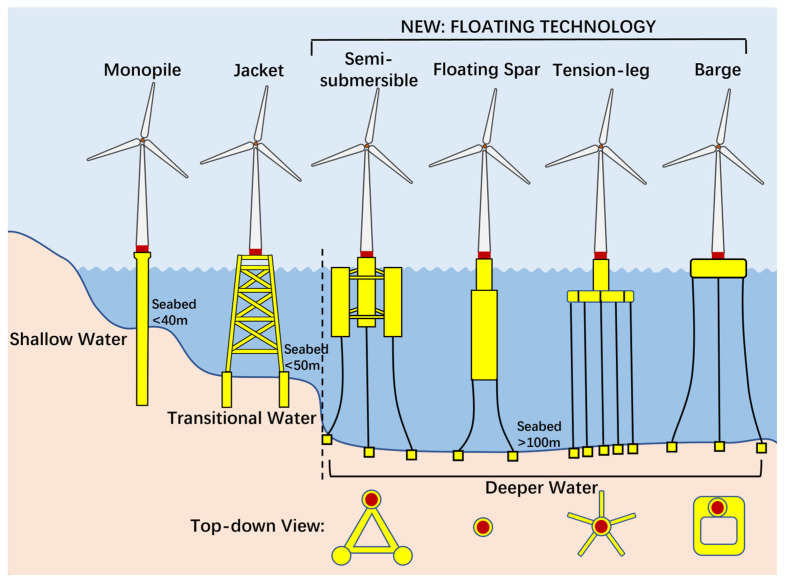
Types of offshore wind foundations are designed to accommodate a range of water depths, spanning from shallow to deep water conditions. (Original figure).

As offshore wind energy development progresses, the need for floating platforms to support wind turbines becomes increasingly apparent, particularly in deeper waters where fixed foundation structures are not feasible but where there are more abundant wind resources, as shown in Figure 2 [7]. Numerous reviews have been undertaken to examine the design challenges pertaining to FOWTs [8,9,10,11,12,13,14,15,16,17,18]. The semi-submersible FOWTs [13,19,20], which are the primary focus of this paper, offer a robust, flexible, and cost-effective option for the offshore wind industry. They have the potential to be installed in locations that allow for stronger and more consistent wind power in large ocean areas with water depths of 100 m or more, making it easier to deploy wind turbines with longer blades [21,22]. However, the geographical positioning and floating nature of the FOWTs within harsh and isolated environments render them susceptible to severe climatic conditions and then break down, thereby presenting considerable challenges in terms of O&M activities. Therefore, in the pursuit of affordable wind energy, it is imperative for researchers to explore techniques aimed at mitigating O&M costs of FOWTs.

### 1.2. Importance of O&M

**Figure 3:**
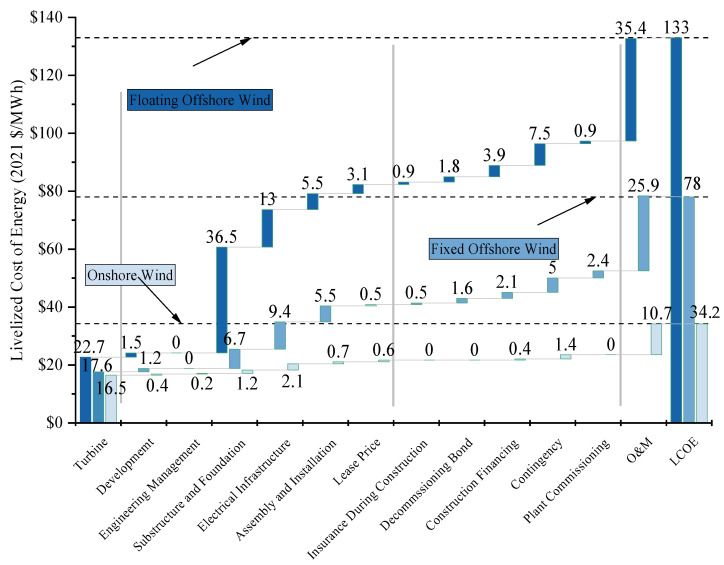
Reference plant levelized cost of energy (LCOE) component cost breakdown of different types of wind turbines (Original figure. Data from Refs. [25,26]).

O&M has been acknowledged as a primary factor in the comprehensive expenditure, typically comprising approximately 25–30% for onshore wind [5] and more than 30% for offshore wind [23], as shown in Figure 3 of the costs incurred throughout their over 25-year-long lifecycle, making it a key area of cost reduction for wind energy if it wishes to compete within the same market as other energies sources [24]. It could be found from Figure 3 that the cost of inspection for FOWTs is much higher than for onshore wind because the onshore wind is mature, and the distance to shore and water depth have a significant impact on the accessibility of maintenance personnel and equipment [1,24,25,26]. The wind turbine O&M market size is poised to grow from USD 13.22 billion in 2022 to USD 33.85 billion by 2030, growing at a compound annual growth rate (CAGR) of 12.71% in the forecast period of 2023–2030 [27], which means that the research in this field will have broad prospects.

Wang et al. [8], Henderson and Witcher [9], and Ren et al. [28] identified O&M as a key area for future research, typically involving regular inspection, maintenance, and repair tasks performed to prevent breakdowns, optimize energy production, and extend the lifespan of the turbines. Inspection activities are crucial to O&M as they are the basis for fault detection and prediction, performance evaluation, and maintenance strategy formulation and can ensure safety compliance and early detection of potential issues, all of which collectively optimize performance and minimize costly downtime. Therefore, the application of more advanced inspection technologies can effectively increase the service life and work efficiency of FOWTs and create huge economic value [29]. The technical feasibility of developing inspection of FOWTs is not an issue as offshore oil and gas have demonstrated the long-term survivability of offshore floating structures. However, it is uneconomic and unsafe to transfer offshore and fixed onshore technology directly to the FOWTs without adaptation [30]. There are no publicly documented commercial applications on inspection for FOWTs due to the limited research on FOWTs [31]. However, some key technologies in inspection missions in other industries, such as infrastructure construction [32], onshore wind power [33], the power industry [34,35], offshore oil and gas [36], etc., could also provide some inspiration and technical support for the future research of FOWTs.

Several literature reviews present the current status and future trends for the inspection of offshore wind turbines, including FOWTs [37,38,39]. Traditional inspection methods include manual inspections, which can be dangerous, time-consuming, labor-intensive, subjective, and expensive, ultimately increasing the levelized cost of electricity. To conduct FOWTs inspection, typically, two to three technicians will travel more than two hours from the harbor using a workboat. Alternatively, a helicopter can be employed to minimize travel time. The inspection process at the site encompasses scrutinizing the platform, tower, hub, and each blade, a task that may require 6–10 h to several days [23,40]. Therefore, it is imperative to explore the possible deployment of remote and autonomous robotic systems in such inspection activities to reduce the expenses associated with human visits. The integration of robotic systems can play a pivotal role not only in replacing monotonous tasks and reducing the levelized cost of energy but also in mitigating health and safety (H&S) hazards by assisting human operators in conducting necessary inspections. For example, the challenging environmental conditions expected at FOWT sites are likely to benefit from unmanned inspections through the use of UAVs due to accessibility issues [24].

### 1.3. Classification of UAV

UAV is an aerial drone-carried robotic system as a payload able to acquire specific datasets like images, 3D points clouds, or other physical parameters like vibration, temperature, or radiation, allowing human beings to reach places that were previously hard to reach for efficiency and safety. As an enabling technology, UAV has been deeply involved in human life and production. UAV represents a safe, cheap, and fast solution for the on-demand acquisition of detailed information. The use of UAVs has progressively increased in the last decade and nowadays started to be considered a standard research instrument [41]. And the current definition of UAV has far exceeded the traditional definition of drones in the past. It is also identified as an unmanned aerial system (UAS).

**Figure 4:**
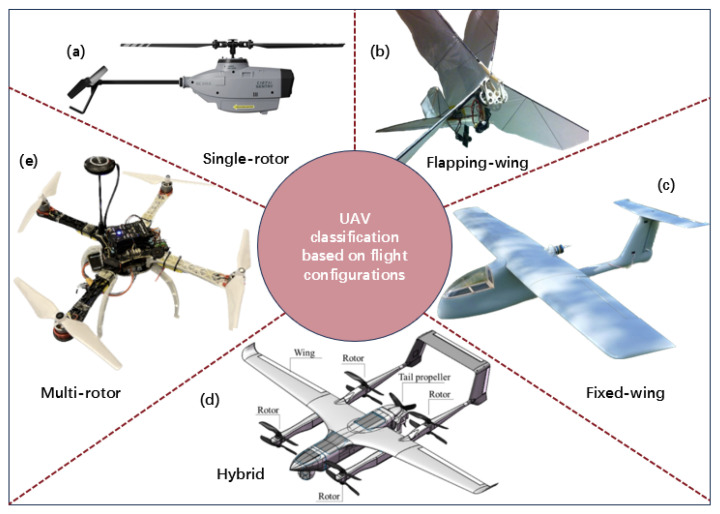
Classification of UAV. (**a**) Single-rotor (helicopter); (**b**) flapping wing (DelFly II), adapted with permission from Ref. [57], copyright ©Clearance Center, Inc.; (**c**) fixed-wing UAV, adapted with permission from Ref. [42], copyright ©2021 MDPI; (**d**) hybrid, adapted with permission from Ref. [58], copyright ©2021 MDPI; (**e**) multi-rotor (quadrotor) UAV.

UAVs can be classified based on several factors, such as size, mean takeoff weight, flight configuration, purpose, power sources, sensor type, autonomy level, etc. [42]. Based on flight configurations, UAVs can be categorized into (see Figure 4): single-rotor (helicopter) [43,44], multi-rotor (tri-rotor, quadrotor, hexacopter) [45,46,47], fixed-wing [48,49], hybrid [50,51], flapping wing (ornithopters and entomopters) [52,53,54]. Multi-rotor UAVs, as one of the most significant members of the UAV family, complement the Internet of Things (IoT)-based techniques with advantages including ease of operation, simple structure, slower speeds, ability to maneuver, mature theory, and relatively low cost [55]. They can facilitate data acquisition at temporal and spatial scales at low costs that remain unachievable for traditional remote sensing platforms [56]. Therefore, multi-rotor UAVs account for the main part of the application of UAVs in industry and science research. So, the discussion of the development and applications of UAVs in this paper is particularly focused on multi-rotor UAVs, and the UAV mentioned later refers to the multi-rotor UAS if there are no additional instructions.

### 1.4. Existing Literature Reviews of Infrastructure Inspection Using UAVs

When searching for existing reviews, only those that reviewed the inspection methods that have the potential to be transferred to FOWTs are considered. Table 1 presents a comprehensive review of research exploring the application of robotics and IoT technology in the inspection of wind turbines and related infrastructure over the past decade, organizing articles by their publication year. These overviews aim to survey the developments made in the past toward the inspection of FOWTs and some other marine infrastructures through alternatives to manual inspection. Before 2020, there was limited exploration using UAVs for testing FOWTs, primarily due to the early stage of commercial FOWT projects. UAVs underwent significant advancements in industrial usage during this period, finding applications in established sectors like construction, power transmission, and oil and gas extraction. Even though UAV technology has a strong foundation, its initial application primarily focused on the traditional energy sector. However, the adoption and integration of this technology into the emerging sustainable energy field, specifically FOWT, typically encounter a slower pace of transfer and implementation. Post-2020, the emergence of commercial FOWT projects spurred the demand for efficient inspection methods, leading to a gradual shift of UAV technology toward FOWT inspection. Additionally, advancements in computer technologies like artificial intelligence and deep learning have further facilitated the integration of UAVs in this domain.

A few review articles mentioned the advantages and great prospects of UAVs for FOWT inspection. Kapoor et al. [59] discussed research work concerning the employment of UAVs as a facilitator for structural health monitoring to accommodate the diagnosis of the state of FOWTs. They emphasize the potential technological impact of the UAVs’ engagement on the information and integrity management of infrastructures for both societal and industrial benefits. Khalid et al. [38] discussed the growing interest in UAVs for conducting inspection and remote sensing tasks in FOWTs, including the characteristics of UAVs relevant to remote inspection in the work, such as their motion capabilities, payload limitations, and communication requirements. Nevertheless, a thorough and all-encompassing review in this field is lacking. To enhance the ability to consolidate and forecast the implementation of UAVs in the inspection of FOWTs, it is imperative to gain a comprehensive understanding of the distinguishing features and prevailing trajectory of UAVs.

At the time of writing, there has been no review article specifically on the inspection of FOWTs using UAVs. Robert et al. [40] presented a survey on the challenges and opportunities of utilizing small, uncrewed aircraft systems to inspect wind farms in their review paper. But, the paper is focused on the case study of commercial applications of UAVs to guide wind farm planning and accurately assess the efficiency and productivity of wind farms by measuring the atmosphere around the turbines rather than the turbines themselves. So, this paper serves as a meta-review by analyzing and comparing existing works, synthesizing their methods, and offering a comprehensive overview of the field. Different from previous review works, this paper will focus on fault detection by acquiring physical signals from UAVs equipped with different types of sensors by introducing the development of UAVs and the principle and application of different sensors. Exploring the existing literature, projects, and technologies offers a valuable foundation for future researchers and can hasten the implementation of UAV inspection in FOWT. By consolidating insights from various disciplines and industries, this study identifies potential research gaps and points toward future directions, ultimately fostering sustainable development of the world. However, it is important to note that because we cover a broad range of research fields, the overview may not encompass every aspect of the subject.

**Table 1:** Summary of the existing literature reviews of the robotic inspection methods of infrastructures sorted by year. USV (unmanned surface vessel), AUV (autonomous underwater vessel), ASV (autonomous surface vessel), and AGV (automated guided vehicle).

Reference	Inspection Method	Target	Mission	Year
[60]	Permanent sensor	Onshore and offshore wind turbines	Health monitoring	2015
[36]	ROV, AUV, AGV, ASV, UAV	Offshore oil and gas	Explorations Inspection Welding Oil spill	2016
[61]	UAV	Building	Inspection	2018
[62]	UAV	Mining area	3D modeling Land damage assessment, geological hazards	2019
[63]	UAV	Bridge	Quantify damage using images captured from UAVs	2020
[64]	UAV	Onshore and offshore wind turbines	Non- destructive testing	2020
[65]	UAV	Large infrastructures	UAV-based NDI of industrial and commercial facilities	2021
[59]	UAV	Civil infrastructure as well as industrial facilities and power plants	Structural health monitoring and management	
[66]	ROV, AUV, UAV, climbing robot	Offshore wind turbines	Robot-based damage assessment	2022
[67]	UAV, USV, AUV, ASV, AGV, climbing robot, quadruped robot, railed robot	Offshore wind turbine	Robotics and artificial intelligence	2022
[38]	ROV, ASV, UAV, climbing robot	Floating offshore wind turbines	Applications of robotics for O&M	2022
[68]	AUV, ASV, UAV	Offshore wind turbines	Collaborative unmanned vehicles	2022
[69]	UAV, climbing robot	Onshore and offshore wind turbines	Non-destructive testing	2022
[70]	UAV	Tunnel	Localization and navigation of UAVs in underground	2023
[40]	UAV	Onshore and offshore wind farm	Atmosphere measurement	2023

### 1.5. Scope of the Review

Compared to other previous review work related to the implementation of UAV inspection, this paper has the following groundbreaking contents:

1. Emphasizing the importance of reducing O&M costs for the FOWT industry;
2. Examining previously conducted projects in different industries that could potentially be applied within FOWT inspection operations;
3. Assessing the prospect of UAV usage by analyzing the long and short advantages of different methods;
4. Introducing the working principles of RGB cameras, thermal imaging sensors, light detection and ranging (LiDAR), ultrasonic sensors, and their functions and applications in inspection. Additionally, introducing the status and prospects of applying these sensors to FOWT inspection;
5. Introducing the development history and characteristics of UAVs and providing the future research direction of UAVs in the field of FOWT.

For the identification of the relevant literature, a comprehensive set of keywords was used in the search strategy. These keywords include condition monitoring (CM), UAV, drone, fault diagnosis, wind turbine, infrastructure, and robotics, among others. The search spanned the past decade to ensure the inclusion of recent developments in the field. Additionally, for infrastructure-related topics, except for wind turbines, the citation timeframe was extended to cover the last 20 years. During the selection of citations, my criteria were broad and encompassed various factors, such as the presence of field experiments and the potential practical applicability of the studies. This multifaceted approach aimed to ensure a thorough and comprehensive review of the relevant literature and projects in the specified domain.

The rest of the article is structured as follows: Section 2 identifies various fault types in FOWTs and presents a comparison of current methods and technologies. Moving forward, Section 3 explores how UAVs have been developed and used in FOWT inspection, summarizing relevant papers and projects from the last decade. Section 4 delves into the challenges and discourse surrounding the potential of multi-robot systems and aerial manipulators as avenues for future research. Lastly, Section 5 encapsulates the conclusion and further discussion.

## 2. Inspection of FOWTs

FOWTs, which have been deployed on a commercial scale since the 2020s, are still in the early stages of development. It is not clear when the existing capacity will complete its technical life or what will happen after that [2]. They are just at the beginning of their service, and the investment for O&M will be an important and ongoing expense during their long technical life. Any improvement in O&M technology will result in continued cost savings throughout the life of the turbines. There is currently a transition occurring toward automation and digitalization, driven by technological advancements, in the O&M of FOWTs [37].

### 2.1. Fault Types and Fault Detection Techniques of FOWTs

FOWTs need to rotate for a long time under various environmental conditions as a complex rotating machine, and each component bears a high load and risk of faults. Especially the rotor and drivetrain, which are subject to continuous rotation and wave exposure, leading to frequent failure rates caused by operational wear and fatigue. Additionally, certain failures are deemed to occur randomly, lacking explicit trends or predictability [71]. The principal failures of the components of wind turbines are enumerated in Ref. [28] as follows:

1. Rotor and blade: deterioration, adjustment errors, rotor imbalance, corrosion of blades and hub, cracks, and severe aeroelastic deflections [72,73,74];
2. Shaft: shaft imbalance, shaft misalignment, shaft damage, and shaft breakage [75];
3. Gearbox: wear, fatigue, pitting, gear tooth damage, tooth braking, eccentricity of toothed wheels, displacement, oil leakage, insufficient lubrication, high oil temperature, and inadequate lubrication [76];
4. Generator: overspeed, overheating, wear, excessive vibration, rotor asymmetries, bar breaks, electrical issues, insulation damage, slip rings, winding damage, and abnormal noises [77];
5. Bearings: overheating, spalling, wear, bearing shell defects, and bearing damage [78]
6. Nacelle: fire outbreaks and yaw errors [79]
7. Tower: fatigue, vibration, foundation weakness, and crack formation [80].

**Figure 5:**
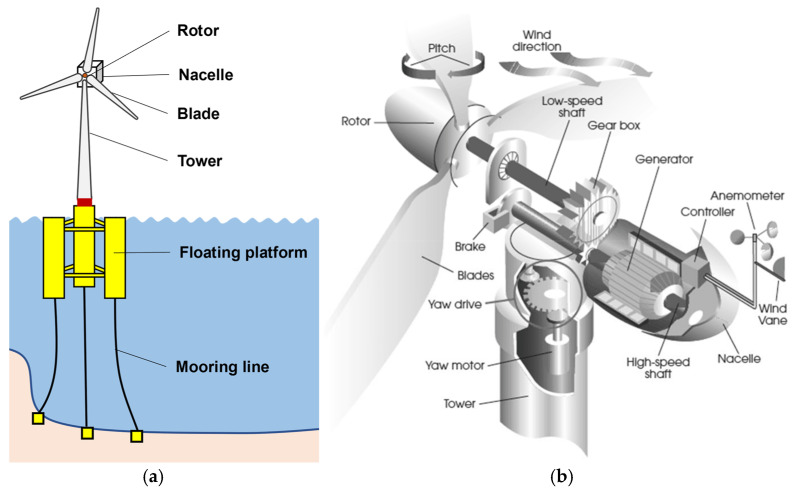
Structure of the FOWT. (**a**) Main components of a semi-submersible FOWT; (**b**) cutaway illustration of a wind turbine nacelle, adapted with permission from Ref. [83], copyright ©2022 MDPI.

Mechanical components always cause a higher amount of downtime when compared to electrical/control ones, reaching more than 75% of the total downtime [81]. Studies have found that approximately 19.4% of wind turbine failures are about blades [73]. Many CM techniques applied to inspect the mechanical components in a wind turbine are listed in Table 2 [28]. It can be analyzed from the table that for FOWTs, the inspection techniques with relatively comprehensive performance include vibration analysis, acoustic emission, and thermography. These techniques are also the most effective fault detection techniques for other rotating machines, such as machine tools and engines. Vibration analysis is a powerful tool in FOWT inspection, which enables the early detection of impending mechanical failure [82]. But, because the acquisition of vibration signals depends on physical contact, it is challenging to obtain reliable vibration signals from different components at high altitudes in harsh environments. Most of the existing inspection methods obtain signals by placing sensors on the site to be inspected manually or by robots. However, it is important to note that because we cover a broad range of research fields, the overview may not encompass every aspect of the subject (Figure 5).

### 2.2. Existing Inspection Methods of FOWTs

**Figure 6:**
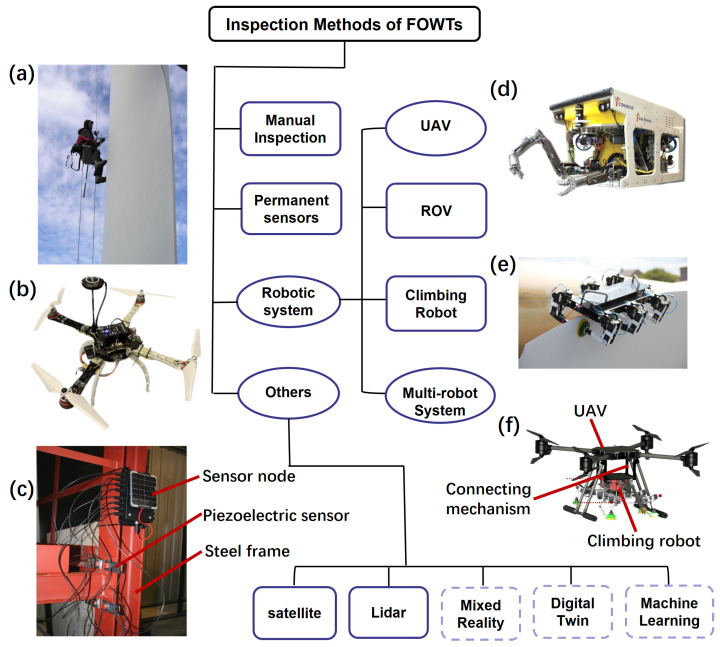
Research pertaining to inspection methods for O&M of FOWTs. Rectangular areas (grayed): widely investigated. Elliptical areas (with filled color): increasing research field. Dotted areas: research gaps. (**a**) The inspectors rope from the rotor and inspects the leading and the trailing edge of blades, adapted with permission from Ref. [84], copyright ©2022 MDPI; (**b**) wind turbine inspection using UAV; (**c**) the wireless sensor nodes were deployed on the steel frame to monitor joints with Piezoelectric sensors to detect the operating status of the wind turbine, adapted with permission from Ref. [96], copyright ©2017 MDPI; (**d**) work class ROV: MRE ROV by the University of Limerick, adapted with permission from Ref. [97], copyright ©2018 MDPI; (**e**) BladeBug MKII IMR robot, adapted with permission from Ref. [98], copyright ©2022 Wiley Periodicals LLC; (**f**) the multi-robot system for wind turbine inspection, adapted with permission from Ref. [98], copyright ©2022 Wiley Periodicals LLC.

Figure 6 shows the existing mainstream FOWT inspection methods, including manual [84,85,86], permanent sensors [60,87], ROV [88,89,90], climbing robot [91,92], UAV [93,94], and so on. The grayed rectangular areas represent sufficient research and successful commercial application examples in this field, while the elliptical areas with filled color represent that this field is receiving increasing attention and the research heat is rising. The dotted areas indicate that the research in this field is still in its infancy and there are many research gaps.

Traditional manual approaches to structural inspections always rely on visual inspection techniques through telescopic lenses, by lift or climbing (including maintenance and repair) [85,86]. Conducting these inspections is often technically intricate, particularly when it comes to examining critical structural elements and hard-to-reach hot spots. This traditional approach typically results in lengthy turbine downtime, leading to a significant reduction in energy production and extensive reliance on crew transfer vessels, which significantly contributes to the overall operational and maintenance expenses of wind farms [95]. At the same time, it also poses severe challenges to the H&S of maintenance personnel. Therefore, in related industries, it is the general trend to reduce human labor by using permanent sensor systems and autonomous robotic solutions; its purpose is to reduce costs and control risks.

Permanent sensors are one of the common means of CM systems, which are commonly utilized for monitoring the primary components of wind turbines [60,99,100,101,102,103,104]. Permanent sensors are employed to collect both environmental conditions (e.g., wind speed, relative humidity, ambient temperature, and turbulence) and device information (e.g., rotor and generator speeds, lubrication state [105,106], currents and voltages, generated power, or downtime due to failure [107]). However, CM instrumentation can include thousands of sensors, which causes additional capital costs due to the purchase, installation, and maintenance estimated at EUR 20,000 for a single FOWT [108]. These systems need to exhibit resilience, possess lifespans that match or exceed those of the wind turbines themselves, and maintain low rates of false alarms for fault detection [60,99]. In certain instances, engineers may even need to develop another monitoring system for permanent sensors themselves, resulting in a significant rise in maintenance costs. An established technique for permanent sensors involves the utilization of piezoelectric accelerometers, which are affixed to either the component or its casing. These sensors can gather dynamic attributes, such as vibration velocity, which can be analyzed for the purpose of monitoring the system [106]. Tchakoua et al. [109] provided a general review and classification of wind turbine CM methods and techniques with a focus on trends and future challenges. Yang et al. [99] summarized the development status and future demand of the existing CM systems and addressed the technical and commercial challenges, in particular, reliability and value for money. Hu et al. [87] proposed a seismic-based method using transfer functions that connect accelerations in the drivetrain and tower to unique seismic signatures, rather than traditional methods that require the deployment of a range of additional sensors. Moreover, instantaneous spectral entropy is a novel signal-processing technique employed for the time-dependent detection of damage events in wind turbines. Marco et al. [110,111] conducted retrospective damage event detection using vibration time histories obtained from a wind turbine gearbox. This approach holds promising potential for widespread utilization in the real-time monitoring of FOWTs in the future.

In the era of permanent sensor deployment, addressing battery-related challenges is crucial. To power low-consumption sensors efficiently, direct energy-harvesting methods have emerged. These devices play a vital role in ensuring uninterrupted power for sustained data collection, eliminating the need for frequent battery maintenance, and contributing to environmental sustainability [112]. Piezoelectric ceramics are widely employed in energy-harvesting devices, leveraging their capability to convert mechanical vibrations into electrical energy, particularly enhancing efficiency in offshore wind energy detection systems and ensuring sustainable power support for fixed sensors while reducing the need for frequent energy interventions [113]. Robotic systems offer the possibility of automating various aspects of O&M, resulting in the continuous acquisition of comprehensive data, improved operational efficiency, and advancements in health and safety. Omer et al. [114] explored the expenses and advantages associated with integrating robotics into the O&M processes of a FOWT using a bottom-up cost model.

ROVs served as underwater robots, capable of performing tasks and collecting data while being operated from the safety of the water’s surface from 100 m depth to several thousand [115,116]. The community has seen great advancements and developments in both theory and applications of ROVs due to the numerous innovations in supporting technologies in energy [117], perception [118], navigation [119], communication [120], control [121], and autonomy [122]. For the research of underwater vehicles, there has been sufficient review and research to summarize their development, application, and challenges, as well as to make a full explanation of their application prospects and advantages and disadvantages [123,124]. Due to technical limitations such as materials and chemistry, ROV endurance will not be able to meet the requirements of long-term independent unmanned work for some time in the future, requiring manual and logistics vessels to continuously carry out launch and recovery. Secondly, the involvement of both manual and logistics vessels means that the ROV operations will be significantly impacted by adverse weather conditions. Strong currents, high waves, or poor visibility can limit the deployment and effectiveness of ROVs, potentially delaying or interrupting inspection activities. A suitable solution is to combine the ROVs with other unmanned robots such as autonomous surface vehicles (ASVs) and UAVs. Zhao et al. [125] examined the hydrodynamic performance of an unmanned vehicle system consisting of an ASV and a ROV. It aims to contribute to the development of a fully autonomous ASV/ROV system for inspection and maintenance missions.

Climbing robots have significant potential in the inspection of FOWTs in visual inspections, structural integrity assessment, non-destructive testing (NDT), and so on. Climbing robots can navigate the complex surfaces of FOWTs to perform visual inspections. They can assess the condition of critical components such as the tower, platform, cables, and connection points. Numerous climbing robots designed for the purpose of inspection have been introduced, with a significant proportion drawing inspiration from reptilian [126], mammalian [127], and insectoid [98] forms. They employ a variety of locomotion modalities, such as climbing and sliding. Liu et al. [92] presented the design and development of a climbing robot for wind turbine inspection and maintenance featuring a unique winding mechanism that allows it to grip the tower surface securely, enabling it to work at significant heights and withstand static and dynamic conditions. Nonetheless, the utilization of these robots necessitates intricate mechanical designs and intricate analysis of dynamics. Furthermore, their applicability is restricted to structures characterized by specific shapes and surface materials. Moreover, the setup time required and the relatively slow climbing speed of these robots result in inspection tasks often demanding a substantial amount of time [128].

UAVs have great potential in the detection of FOWTs, which will be discussed in detail in Section 3. In addition to the above, there are several technologies that can be used for the inspection of FOWTs, such as soft sensors [129,130], mixed reality [131], digital twin, data mining [132], IoT [133], machine learning [134,135], satellite imaging [62], and multi-robot system, which will be introduced later.

### 2.3. Comparison of Different Methods

This section will analyze the performance of different methods in security, economy, efficiency, automation, and endurance time. FOWTs are engineered structures vulnerable to high wind conditions, featuring numerous intricate mechanical and electrical components. Consequently, it is imperative to cultivate an understanding of the potential hazards encountered by researchers in wind turbine operations [33]. This increased risk not only creates H&S problems for workers but also affects the efficiency of wind farm operations and increases maintenance costs. In addition, in order to ensure safety, risk control measures have to be taken, which also increases the cost expenditure.

One of the established approaches employed for the purpose of ranking or prioritizing risks is the employment of a risk matrix, as depicted in Table 3 [136]. In this matrix, the vertical column represents the probability or likelihood of the occurrence of a given risk, while the horizontal row illustrates the level of severity associated with its potential manifestation. Depending on the degree of danger, the horizontal rows and vertical columns are assigned a number from 1 to 5, with higher values representing higher probability or severity. The risk ranking of the event is obtained by multiplying the values of the corresponding columns or rows in the table. The risk assessment of different methods is analyzed, and the most serious results of each method are summarized in Table 4. Methods that minimize risks and increase safety will result in long-term cost savings.

Unlike manual inspection, the cost of permanent sensors and robotic inspection methods is mainly the initial investment cost, including the cost of equipment, infrastructure, training, and setup. The expenses associated with purchasing robots, sensors, and vehicles can be reused to maximize economic value. However, the manual inspection method means that the same personnel salaries, insurance, and other costs must be spent every time. Meanwhile, manual inspection may require many trained personnel to physically inspect and analyze data, while climbing robots, ROVs, or UAVs may need fewer operators or pilots to control the devices. Considering the associated labor costs and any necessary training or certification requirements, the cost performance of manual detection is the lowest. Considering the potential duration of non-operational periods necessary for inspection or maintenance purposes, the repercussions of such downtime on productivity and its corresponding financial implications should be evaluated. Techniques that necessitate regular maintenance or possess lengthier repair intervals might lead to heightened instances of non-operational periods and augmented economic expenditures.

UAVs perform well in this regard because of their great flexibility and fast speed. On the contrary, manual inspection has the worst economy due to high labor costs and safety risks. A study [137] of an offshore wind farm combines both financial and physical models using techno-economic analysis and activity-based costing, utilizing data from various sources in the open literature. Specific UAV operational models are created, drawing from expert knowledge in operational practices and predicting environmental conditions. Initially, rope-access inspection serves as a foundational method, constituting 0.7% of the wind farm’s operational expenses. Substituting UAVs for rope-access inspection demonstrates a potential cost reduction of 70% and a substantial decrease in revenue loss, estimated at up to 90%, due to minimized downtime. ROVs and climbing robots are economical over permanent sensors because they can perform the same task repeatedly in different locations.

Ranking the efficiency of inspection methods for floating wind turbines would involve considering and weighing the speed and accuracy. Each method may have advantages and limitations based on the specific circumstances of the wind farm and the inspection requirements. The duration of inspection methods for floating wind turbines can vary depending on several factors, such as the size and complexity of the turbine, the specific components being inspected, the accessibility of the site, and the extent of the inspection. The duration of manual inspections can vary significantly depending on the size of the turbine and the scope of the inspection. It can range from a few hours to several days or even weeks for more comprehensive inspections. The time required includes setup, safety procedures, visual inspections, and data collection. The installation of permanent sensors and structural monitoring systems can take several days to weeks, depending on the number of sensors and the complexity of the system. Once installed, these systems can continuously monitor the turbine’s condition, providing real-time data over an extended period. Climbing robot inspections typically require more time compared to other robotics methods. The duration can range from several hours to multiple days, considering setup, climbing, data collection, and any necessary maintenance. The duration of ROV inspections can typically range from a few hours to several days, considering the time required for deployment, maneuvering, data collection, and analysis. UAV inspections offer relatively quick and efficient data collection. Typically, UAV inspections can range from a few hours to a day, including flight planning, data collection, and post-processing.

The ultimate purpose of remote and autonomous inspection of FOWTs is to be completely able to carry out missions/tasks with minimum human interaction and supervision. For UAVs to attain widespread utilization as aerial imaging or sensor-based instruments accessible to domain specialists rather than exclusively trained pilots, there is a fundamental need to enhance their level of autonomy [128]. Different levels of autonomy of robotic systems can be achieved toward that goal depending on the complexity of tasks, the severity of the environment, and whether a fully autonomous solution exists or not for that specific application [42]. These levels were defined by many researchers and organizations. The National Institute of Standards and Technology of the U.S. defined the levels as the mode of operation for unmanned systems (UMS) depending on the human operator’s ability to interact with a UMS to perform the operator-assigned missions as follows [138]:

Fully autonomous: The robotic system can adapt itself to the environment without human intervention and accomplish its assigned mission.

Semi-autonomous: The robotic system is capable of autonomous operation but requires various levels of human–robot interaction/interface.

Teleoperation: The human operator controls the actuators remotely using sensory feedback.

Remote control: The human operator controls the actuators continuously and remotely on a continuous basis without any initiative of the robot.

For manual inspection, the human operator directly controls the inspection process without any autonomy or automation. Permanent sensors, being stationary and continuously collecting data without human intervention, operate fully autonomously. Climbing robots exhibit a certain level of autonomous operation, although occasional human intervention or guidance may be required, rendering them semi-autonomous. Consequently, they possess a degree of autonomy. ROVs are classified as teleoperation systems since they are operated remotely by a human operator who controls the vehicle through sensory feedback. The degree of automation in UAVs is notably intricate, with research and applications encompassing a broad spectrum from teleoperation to full autonomy. UAVs have the capability to function either in a fully autonomous or semi-autonomous mode, wherein they can perform tasks independently with or without human intervention, or in a teleoperation mode.

Endurance time refers to the duration or length of time that a particular method or device can operate continuously without requiring recharging or refueling. The endurance time of the manual method heavily depends on the physical stamina and endurance of the personnel conducting the inspection, typically ranging from a few hours to a full day. The endurance time for permanent sensors is considered high, as they can operate for extended periods, sometimes even years, without interruption. The endurance time of climbing robots and ROVs usually varies depending on factors like battery capacity, energy efficiency, and the complexity of the inspection task. There are also robots that can be connected to a base or a support structure through a cable for both power supply and communication purposes. UAVs’ endurance time is significantly based on factors such as battery capacity, payload weight, and weather conditions. Small consumer-grade UAVs often have relatively short endurance times, typically ranging from 20 to 30 min.

Through the above analysis of security, economy, efficiency, automation, and endurance time, the UAV’s superior comprehensive performance is indicated as summarized in Table 5. However, it is crucial to acknowledge its limitations, particularly in relation to battery life and endurance, which present significant challenges but cannot be effectively addressed within a predictable time frame. The ability to prolong the operational duration of UAVs remains an elusive goal, prompting the exploration of alternative research directions to mitigate this issue. Through the synthesis of various approaches, it can be inferred that distinct methodologies possess their own merits and demerits across different facets. Consequently, a singular methodology is typically insufficient to satisfy all the demands of an inspection mission comprehensively. Hence, the amalgamation of diverse inspection methods presents a promising trajectory for future exploration. Considering this, a promising approach is to combine UAVs with other robot platforms, thereby compensating for their endurance deficiencies by leveraging the inherent advantages offered by alternative platforms, which will be introduced later.

## 3. UAV Techniques and Applications in FOWTs Inspection

**Figure 7:**
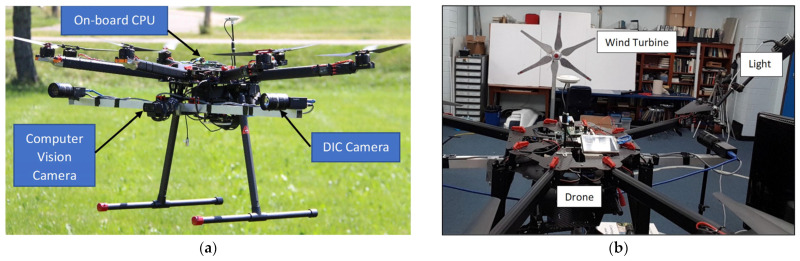
The application of UAVs to inspect wind turbines, adapted with permission from Ref. [93], copyright ©2020 Elsevier Ltd.; (**a**) camera mounted on the UAV for digital image correlation measurements; (**b**) UAV indoor test aims to obtain the dynamic properties of the wind turbine for in situ conditions using a non-contact measurement technique.

Due to their high mobility and simple structure, UAVs can be rapidly deployed and automatically perform repetitive tasks in different locations, greatly reducing the workload of manual labor and saving time and cost. By leveraging UAV technology, operators can enhance the efficiency and effectiveness of FOWT inspections, ensuring the continued O&M of these vital renewable energy assets to produce cheaper energy, promote industry and science, and protect the environment. UAV-based inspection provides a cost-effective and efficient solution by enabling close-range inspections without the need for human presence on the turbines. This approach enhances safety, reduces operational costs, and minimizes turbine downtime by (1) enhanced frequency and expanded spatial coverage of the wind farm within a condensed time frame, (2) the capability to affix diverse imaging and acoustic sensors onto the UAV, enabling comprehensive data acquisition, and (3) the advancement in H&S considerations by alleviating the need for human personnel to access the FOWTs (Figure 7).

### 3.1. UAVs’ Development and Applications in Industries

**Figure 8:**
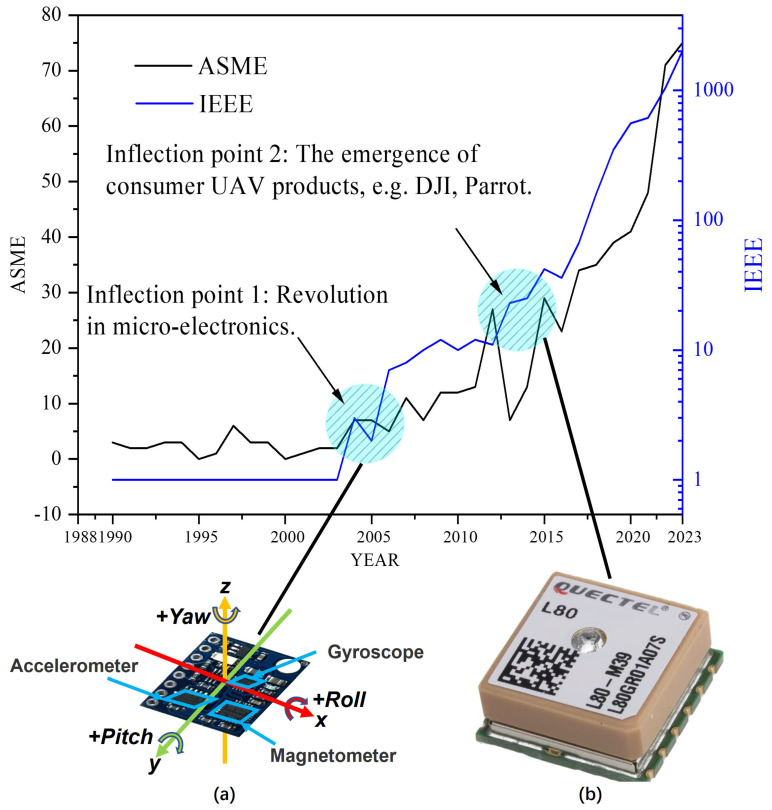
The number of published papers every year during 1990–2023 about UAVs in journals of IEEE and ASME can reflect their inflection points because of the birth of (**a**) MEMS inertial navigation sensor (GY-85), (**b**) GPS module and integrated patch antenna.

As an enabling technology, the development direction of UAVs is guided by task requirements, and it can also use the widespread and versatility of its own applications to inspire and diversify the task. Figure 8a conducts a bibliometric analysis illustrating the number of publications of UAVs in the acknowledged databases, IEEE and ASME, from 1990 to 2022, which reflects the trend in their research and development. It can be found from the figure that there are two significant inflection points in the research trend of UAVs, respectively, in 2005 and 2014. The reason for the first inflection point is that there were advancements in micro-electro-mechanical system (MEMS) technology, particularly in the small and affordable inertial measurement unit (IMU) and GPS receivers, as shown in Figure 8b,c in the mid-2000s [139,140,141]. There was another inflection point in the research trend of UAVs in 2014, which revealed an exponential surge in the number and growth rate of related investigations after that year. Because there was a growing demand for multi-rotor UAVs in civilian applications, which provided the necessary resources and motivation for research and development in this area, the global expenditures on commercial UAVs in 2014 stood at USD 700 million, with DJI being the market leader, followed by Parrot and 3DRobotics [142]. The development of UAVs in 2014 marked a significant breakthrough in the field of aviation.

UAVs have become one of the most vital of the emerging industry. The low price and the very friendly use of commercial UAVs make these systems considered suitable for an incredible number of potential application fields, even where the users are not particularly skilled in aeronautics systems [41]. Many researchers have conducted some comprehensive literature reviews [143,144], summarizing the application status and prospect of UAVs with the benefits over manned systems in terms of mission safety and operational costs. UAVs are gradually used in more and more industries that rely on human labor, especially with a dangerous, repetitive, and wide range of activities, including military operations [145,146], agriculture [55,147], mining [62,148], disaster relief [149,150], surveillance and monitoring [151,152], infrastructure inspection [70], IoT [153,154], domestic law enforcement [155], archaeological excavations [156], film and photography [157], forest fire detection and firefighting operations [158], and so on.

### 3.2. Payload Hardware and Its Functions in Inspection

Early damage identification is crucial for minimizing maintenance costs, mitigating operational uncertainties, and preventing catastrophic consequences [159]. For example, prompt detection of minor turbine blade damage allows for efficient on-site management before it escalates into more significant issues necessitating blade replacement [100]. Subsequently, the replacement of large turbine components entails substantial expenses associated with disassembly, transportation, and reassembly. Numerous methods have been developed to facilitate damage detection in wind turbines. Based on their working principles and distinctive features, on-site NDE techniques encompass vibration analyses [160], ultrasound [161], X-ray [162], strain sensing [163], acoustic emission [164], computer vision [165], thermography [166], eddy current [167], hyperspectral imaging [168], and so on. The different kinds of payloads carried by the UAVs, that is, the various sensors, can give the UAVs the ability to acquire different physical signals.

**Figure 9:**
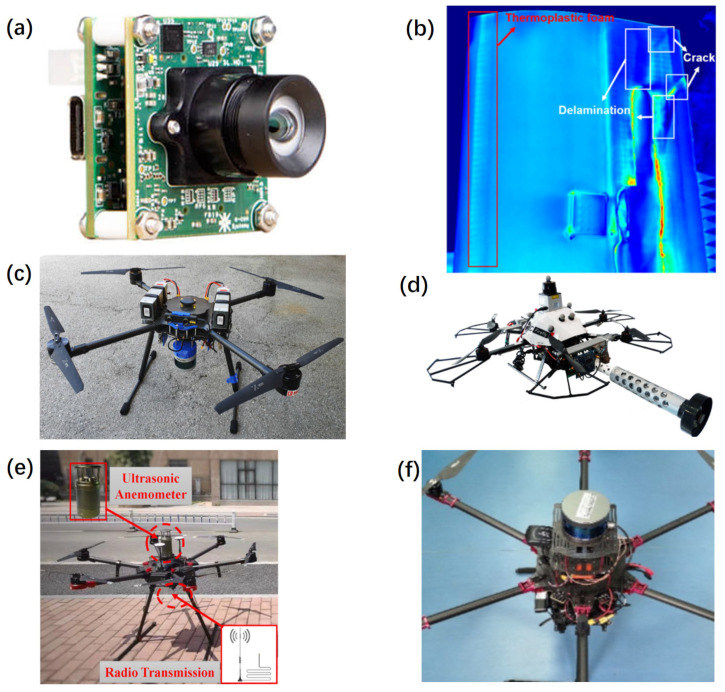
Payload sensors of UAV. (**a**) RGB camera; (**b**) phase images of the passive thermograms captured, adapted with permission from Ref. [166], copyright ©2018 MDPI; (**c**) LiDAR-equipped UAV used for performing wind turbine blade inspection, adapted with permission from Ref. [94], copyright ©2020 IEEE; (**d**) autonomous ultrasonic inspection, adapted with permission from Ref. [178], copyright ©2018 IEEE; (**e**) UAV equipped with ultrasonic anemometer, adapted with permission from Ref. [180], copyright ©2022 Elsevier Ltd.; (**f**) UAV equipped 3 LiDAR, an RGB-D camera, an IMU, an optical flow sensor, a barometer, and a global navigation satellite system (GNSS) receivers, adapted with permission from Ref. [181], copyright ©2020 MDPI.

Currently, UAVs primarily transport advanced high-definition cameras to inspect turbine blades. Thanks to advancements in optics and computer tech, visual monitoring has evolved from static detection to dynamic surveillance. RGB cameras (shown in Figure 9a) can capture high-resolution images for visual inspection of the turbines, assisting in identifying surface defects, corrosion, or other anomalies. Moolan-Feroze et al. [169] presented a method for simultaneously localizing a UAV and fitting a wind turbine model for surface inspection. However, the distance resolution of the visual system is limited to a mere four meters per pixel, resulting in a comparatively lower precision when compared to a LiDAR-based system. However, ensuring continuous focus of the camera throughout the entirety of the inspection procedure entails maintaining a consistent relative proximity to the blade, which frequently presents challenges in adverse atmospheric conditions at elevated heights.

However, the quality of image-only inspections is often affected by the motion blur of UAVs for visual inspection and damage detection on civil structures. The quality of photographs and videos captured through the utilization of UAVs is significantly impacted by a myriad of factors, including but not limited to lighting circumstances, proximity to the subject, and the motion of the vehicle arising from environmental influences [170].

Deep learning techniques like support vector machines and convolutional neural networks have significantly enhanced the automatic extraction of intricate blade damage features, marking a notable advancement in this field [74]. Multiple studies have explored using machine learning methods to automate the analysis of wind turbine inspections, aiming to reduce both time and expenses [65]. Shihavuddin et al. [171] pioneered an automated damage detection system using deep learning, employing meticulously labeled data. Their approach involved utilizing a dataset captured by an RGB camera mounted on a UAV featuring diverse wind turbines in Denmark. This dataset was used to both train and evaluate their algorithm. They employed a fast R-CNN deep network, leveraging both manually annotated samples and expanded data to effectively identify defects. The system primarily offered guidance to inspectors during data analysis.

Some visual inspection missions aim to gather high-resolution visual data pertaining to specific areas of interest and to generate three-dimensional surface models. Subsequently, inspection experts will utilize all accessible data to scrutinize and identify potential flaws in their assets. Kanellakis et al. [172] utilized a monocular camera structure from motion [173] to process captured data and create a global representation. The approach is chosen for its scalability and ability to capture depth information. The collected data are downsampled to remove redundancy, and multi-view stereo algorithms [174] are used for dense reconstruction based on stereo image pairs.

Infrared thermography (IRT) (shown in Figure 9b) stands out as an NDT with the capability to detect the most significant defects that may arise during the service life of composite wind blades, such as cracks, voids, delamination, structural damage, and corrosion, by observing the component’s thermal radiation patterns [65]. IRT provides detailed visualization of internal as well as external blade damage when compared with inspections completed by the naked eye. Galleguillos et al. [175] presented an NDT method for the inspection with an IRT and examining blades on-site. The presented inspection methodology could effectively detect flaws in the turbine blades, such as cracks, impacts, and delamination. This study verified that ambient variations in temperature are sufficient for revealing defects through IRT. By using UAVs and thermographic imaging, the time of wind turbine inspections is reduced to 15–20 min per blade, and risk to human inspectors is minimized.

LiDAR (shown in Figure 9c) is used to measure distances and generate precise 3D models of wind turbine blades, helping to detect deformities or damage. LiDAR sensors possess a noteworthy capacity to gather a substantial quantity of precise point coordinates over considerable distances, measuring distances and generating precise 3D models of wind turbine blades, helping to detect deformities or damage. The convergence of these sensors with UAVs presents a formidable synergy, enabling the pursuit of more intricate undertakings. Schäfer et al. [176] proposed a concept for a UAV to perform automated flight using 2D LiDAR at a wind turbine with the potential to improve safety, efficiency, and cost-effectiveness with a priori 3D mapping of the plant, spline-based flight path planning, and a collision avoidance and distance control system. Car et al. [94] demonstrated the utilization of a UAV equipped with LiDAR sensors in a semi-autonomous inspection scenario of wind turbine blades. The UAV navigates from the blade base to the tip and returns, maintaining a consistent relative distance and alignment with the blade plane. This approach also enables the acquisition of a comprehensive 3D model of the wind turbine structure.

Ultrasonic sensors (shown in Figure 9d) are employed for thickness measurements and structural integrity evaluation of turbine components, particularly the tower and blades. Nearly all the current research on using UAVs in FOWT inspection is based on computer vision and image analysis other than capturing physical signals because it is technically easy for UAVs to take images. Traditionally, the photogrammetric inspection cannot distinguish minute discontinuities or deformations beneath a surface coating. Ultrasonic sensors play a crucial role in assessing crack dimensions and evaluating the structural state of infrastructure. But, ultrasonic techniques necessitate direct contact between the sensor and the surface of the bridge or tunnel, along with the application of a consistent force during measurements [177]. These methods enable precise measurements of crack depth and width, providing valuable insights into the condition of the infrastructure’s components. Zhang et al. [178] presented a contact inspection method with an ultrasound probe installed at the tip of a spring-loaded arm extending from the center of the UAV and simulated a scenario common to industrial inspections.

Anemometers (shown in Figure 9e) measure wind speed, providing crucial data on the wind speeds in the vicinity of wind turbines. Various types of anemometers include cup anemometers and ultrasonic anemometers, among others. Weather sensors encompass sensors measuring temperature, humidity, atmospheric pressure, and wind direction. Such data aid in comprehending environmental conditions, guiding the operation and maintenance of wind turbines. These sensors aid in understanding the dynamic environmental factors influencing the performance and operational status of wind energy generators. Wind resource assessment requires wind measurements on-site for about two years [179]. More measurements produce a fuller picture of available wind resources. UAVs can provide in situ wind and atmospheric state measurements at targeted locations to augment observational records. And continued atmospheric measurement around installed turbines is required for the performance evaluation of a wind farm while providing helpful information that can be used to improve future wind farm projects. In 2022, Li et al. [180] published results from a study on the use of UAVs for detecting wind turbine wakes with a DJI M600PRO drone. The UAV was equipped with a 0.5 kg SA210 ultrasonic anemometer capable of sensing winds at speeds ranging from 0 to 50 m/s. This demonstrates the ability of UAVs to take precise, coordinated measurements around installed turbines.

In the inspection of FOWTs, employing multiple sensors concurrently via UAVs (shown in Figure 9f) is crucial due to the complex and multifaceted nature of the task. Integrating data from various sensors provides a comprehensive understanding of both the turbines and their surrounding environment, contributing to increased efficiency and reliability of wind farms. By amalgamating data from multiple sensors, it minimizes the shortcomings of individual sensors and increases the overall accuracy and reliability of the inspection process. This fusion allows cross-validation and more precise identification of potential issues or anomalies. What is more, the concurrent use of multiple sensors streamlines the inspection process. Instead of conducting separate missions for different types of inspections, a single UAV equipped with multiple sensors can gather various data types in one flight, saving time and resources. Sa et al. [128] presented a shared autonomy approach using UAVs to inspect vertical pole-like infrastructure. The proposed system utilizes an image-based visual servoing (IBVS) technique with two line features to stabilize the UAV and incorporates visual, inertial, and sonar data.

### 3.3. Control of UAV in Inspection

#### 3.3.1. Classification of UAV Automation Levels

Depending on the degree of human participation in the task weight, the application of UAVs in the inspection of FOWTs can be classified into fully autonomous, semi-autonomous, teleoperation, and remote control [68,138]. At present, most of the commercial applications of UAVs in FOWT inspection are based on manual remote control. The operator visually controls the UAV near the blade or tower and then uses the onboard high-definition camera or other equipment to collect the required information. However, manual operation often means that UAVs and FOWTs need to be in the pilot’s line of sight, which means that the pilot and equipment, such as the remote control and ground station, need to be transported to the location of the wind farm. This demand means the expenditure of personnel and logistics vessels but also means that the mission will be affected by weather, sea conditions, working time, and other factors, affecting the efficiency of the inspection and ultimately increasing the operating cost of the wind farm. These limitations further hinder the potential for conducting unmanned inspections for FOWTs [66].

**Figure 10:**
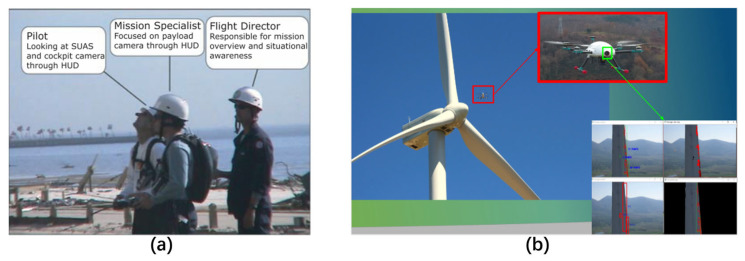
Manually operated and automatic UAVs. (**a**) Flight team with mission responsibilities noted, adapted with permission from Ref. [182], copyright ©2009 Wiley Periodicals LLC; (**b**) WEGOOI project: Inspection services for wind turbines, damage assessment and repair recommendation reports using artificial intelligence and reviewed by engineers, image from https://aleriontec.com/en/project/wegggoi/ accessed on 20 December 2023 (Ref. [184]).

For manually operated UAVs, the application is more embodied in experience, relevant standards, or specifications than in academic publications. The team tasked with executing missions involving near-vertical structures typically comprises three key roles: the flight director, the pilot, and the mission specialist, as shown in Figure 10a [182]. Given their expediency in adverse and high-altitude settings, manually operated UAVs do not exhibit novelty within this domain. The current trend is that newly constructed wind turbines surpass a hub height of 100 m. The manual operational complexities associated with UAV navigation in this particular scenario pose significant challenges. Existing regulations mandate that the operator of a UAV must maintain direct visual observation of the aircraft [183]. Nevertheless, this task poses a considerable challenge for the operator in ensuring a secure and consistent distance from the structure under inspection. Assessing the appropriate standoff distance becomes arduous from the ground perspective, and once the aircraft becomes obscured by the structure, such evaluation becomes unfeasible.

Furthermore, when conducting successive inspections of multiple wind turbines, the repetitive nature of the task can induce fatigue and weariness of the pilot, which increases the probability of hazards, affects the efficiency of the wind farm, and increases operating costs [176]. Meanwhile, instances of communication breakdowns between pilots and the UAVs can potentially lead to a relinquishment of operational command [39]. Automated operation of UAVs reduces reliance on real-time communication, allowing them to operate autonomously and execute pre-programmed flight plans even in the event of communication failures. They enable extended-range and beyond-line-of-sight operations while minimizing the potential for human error. Consequently, some researchers have turned their attention to the automated inspection of UAVs. In recent years, with the development of computer vision, multi-sensor fusion, and other technologies, some automatically operated UAVs for FOWTs inspection technologies and projects have been proposed [85,94,169,172], but all of these projects are in the laboratory or simulation stage, and there is still a certain gap between actual application. Conducting field operations of UAVs in non-laboratory settings, particularly in proximity to expansive infrastructural installations like wind turbines, presents substantial multidisciplinary research challenges.

At present, the research on UAVs’ automatic inspection of FOWTs mainly focuses on navigation, 3D modeling, and defect identification [159] based on computer vision and multisensory fusion by using different types of sensors. The EU-funded WEGOOI project has developed an autonomous wind turbine inspection UAV powered by AI for both onshore and offshore platforms, including software to expand the UAV’s navigation trajectory or path to take in all three blades from four sides in a single flight and the landing strategy allows landing on a moving boat, important for offshore inspections. The goal of this project is to make offshore inspections efficient and fully automated, significantly bringing costs down to half that of other UAV platforms [184]. However, the project is still in the experimental phase. Future work will industrialize the WEGOOI platform from prototype to product. The hardware part needs to be built to maritime environment standards for a robust product that is operational in the wind farm all year round [185,186]. As one significant part of the WEGOOI project, Romain (shown in Figure 10b) aims to develop a complete solution for robotic-based inspection and repair of wind turbine blades. Thermography and stereography with laser heating are integrated so that advanced lock-in techniques can be achieved for up-to-tower inspection of both surface and subsurface defects within blades [184].

#### 3.3.2. Control Algorithm

Developing the application of UAVs introduces several challenges related to control. Firstly, achieving real-time pose estimation, vital for implementing feedback loops, poses significant hurdles. In environments lacking instrumentation, individual sensors cannot directly measure pose, necessitating a fusion of sensor data. Algorithms for data fusion must address sensor limitations, like unreliable magnetometers, imprecise GPS devices, and the limited frequency of visual processing from cameras. What is more, feedback control laws must swiftly respond and maintain stability against aerodynamic disruptions, such as sudden gusts of wind. As introduced before, these complexities are heightened in UAVs due to their restricted payload capacity, heightened susceptibility to aerodynamic disruptions, and the intricacies of aerodynamic effects at this scale.

**Figure 11:**
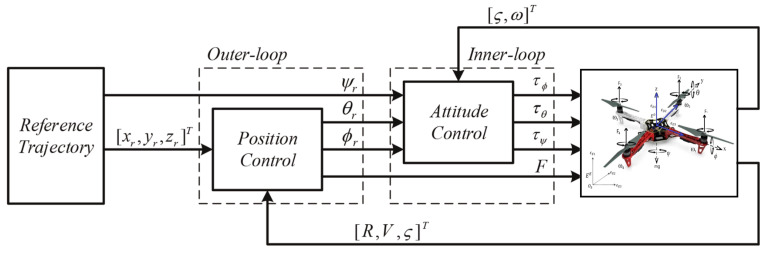
The control scheme of the quadcopter consists of the attitude control loop and the inner loop, which produces the control commands for the quadcopter to move. Moreover, the position control loop and the outer loop produce the references for the inner loop. Adapted with permission from Refs. [190,191], copyright ©2019 Abdulkader et al. & ©2023 MDPI.

Typically, a UAV is tasked with safely navigating along a designated route or visiting specific points of interest to accomplish predefined missions. Máthé et al. [32] separated the control tasks into two primary levels: low-level flight control and high-level flight planning. Primary low-level control duties in UAVs involve three main aspects: achieving flight, stabilizing the UAV, and following a flight route. These responsibilities are managed through attitude and position control mechanisms based on the dynamic model of UAVs, as shown in Figure 11. Attitude control specifically handles flight stabilization and aligning with the intended direction, while position control focuses on tracing and adhering to the planned trajectory. Meanwhile, the latter involves trajectory tracking, which stems from effective position control. Typical low-level flight control algorithms like PD-PID (proportional-derivative-proportional) have demonstrated strong performance [187]. However, certain situations demand more intricate high-level flight control methodologies. For instance, for UAV operations, advanced approaches involving dynamic system modeling become essential [188]. Additionally, algorithms like Cartesian impedance control [189] are employed to tackle specific challenges in control systems. The important applications of high-level flight control systems are localization, navigation, and obstacle avoidance, which will be introduced later. Based on these tasks, there are also extended applications, such as target tracking and simultaneous localization and mapping (SLAM).

Control algorithms ensuring robustness against measurement errors and unforeseen dynamics, such as aerodynamic disturbances, are crucial. The feedback control techniques examined encompass both simple linear methods and advanced nonlinear solutions by Hua et al. [192], covering various methods for stabilizing and controlling systems at lower levels. Linear control techniques rely on approximating a system’s dynamics around feasible state trajectories. Unlike control designs centered on linearization, nonlinear methods can produce controllers with a notably broader stability range and increased robustness. This is particularly evident in highly nonlinear systems, instances of input saturations, or rapid time-varying perturbations. The nonlinear approach to the control problems has definite assets with respect to the well-established and universally used linearization approach. Goerzen et al. [193] focused on high-level planning strategies. Additionally, Dadkhah et al. [194] provided an overview of planning techniques, specifically addressing uncertainties in the process.

#### 3.3.3. Localization and Navigation

Deploying UAVs beyond laboratory settings, particularly in proximity to extensive infrastructural installations like wind turbines, presents noteworthy multidisciplinary research challenges. Among these, the utmost priority lies in developing a precise localization and navigation system that is simultaneously facile to implement. The techniques commonly employed for UAV localization and navigation in inspection operations include GPS, IMU, visual odometry, LiDAR, radio frequency (RF) signal localization, SLAM, machine learning, and artificial intelligence (AI). One of the primary positioning systems utilized for UAVs is the GNSS. However, its reliability can be constrained in certain scenarios. The quality of the GNSS signal diminishes, and at times, it may be completely obstructed near substantial structures. This limitation compromises its dependability in guiding UAVs during their approach and contact with inspection targets.

Visual navigation has been extensively researched for multi-rotor applications, specifically for navigating in environments where GPS is not available and for avoiding collisions. Stokkeland et al. [85] introduced an autonomous machine vision module for UAV navigation in wind turbine inspection, estimating relative position and tracking blade locations using Hough transform and Kalman filter algorithms. Experimental evaluation confirms its accuracy and real-time performance on a UAV’s single-board computer. A significant limitation of this study lies in its exclusive focus on the initial stage of the UAVs’ operation, neglecting any endeavor to estimate the crucial parameters throughout the subsequent and pivotal phases of the inspection process. The weakness of visual navigation lies in its inability to reliably provide localization feedback in high altitudes, especially in the context of visual inertial odometry. In such environments, the lack of distinct features due to feature-poor surfaces, like the flat white color of wind turbine towers, makes it challenging for the software to accurately determine its movement state, leading to convergence difficulties [172].

RF communication uses a wide band of the RF spectrum and pulses to transmit messages accurately and estimate the distance between transceivers. Unlike carrier wave-based radios, ultra-wideband (UWB) radios mitigate multipathing issues and can reconstruct pulses from multiple reflections, resulting in stronger received signals. Kanellakis et al. [172] introduced a novel navigation framework by establishing a team of fully autonomous UAVs with robust localization, planning, and perception capabilities for autonomous visual pre-inspection of wind turbines using the cooperative coverage path planning algorithm. The system achieves high autonomy, fast deployment, and accurate performance using a UWB-inertial estimation scheme. The architecture includes a geometry-based path planner, accurate localization, and visual data post-processing. However, this system also has some disadvantages. Firstly, neither UWB localization nor GPS provides a robust heading estimate, and the wind turbine causes magnetic disturbances that cause the magnetometers to fail. The reliability and location accuracy of UWB transceivers also need to be further verified. Secondly, the deployment efficiency of UAV clusters and UWB networks is much lower than that of individual UAV systems with other localization methods. Thirdly, extra trajectories are necessary to compensate for the disturbance caused by weather conditions.

**Figure 12:**
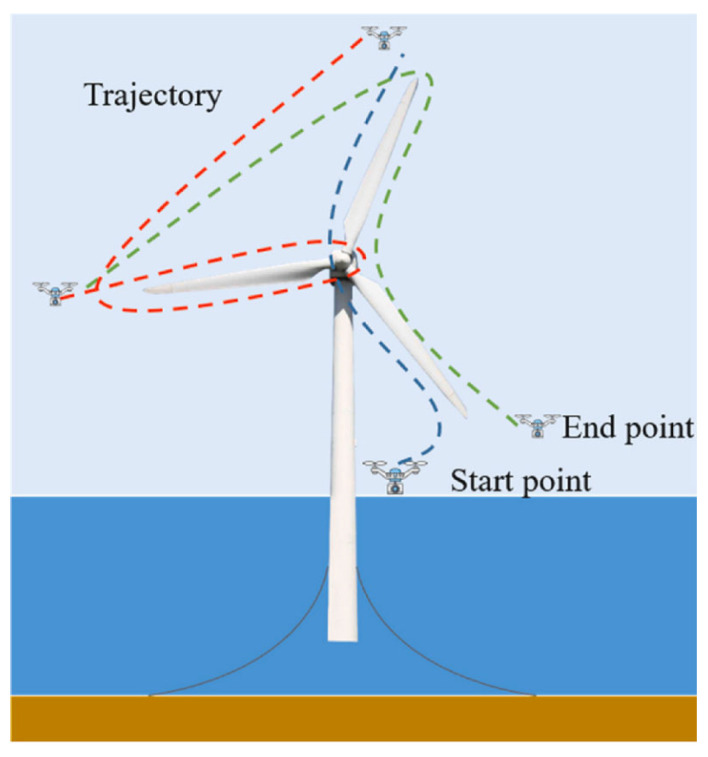
Inspection trajectory of a UAV for FOWT blades and illustration of path planning, adapted with permission from Ref. [64], copyright ©2022 Elsevier Ltd.

What is more, a motion capture system could be utilized to comprise a sequence of cameras positioned within the UAV flight zone. For location and navigation purposes, ultrasonic beacon systems [195] are commonly employed, contingent upon a pre-existing framework set up in the flight area. It is imperative to acknowledge that the advancement of a mature project typically entails not only disparate individual technologies but also the seamless amalgamation of diverse hardware, software, and algorithmic components. Combining multiple localization and navigation solutions can lead to more robust and accurate UAV operations. Given the constraints imposed by limited battery power, the implementation of a robust path planning scheme, as illustrated in Figure 12, becomes crucial in order to optimize the inspection procedure, which poses a challenge for advanced control algorithms.

### 3.4. Applications of UAVs in FOWTs Inspection

Khadka et al. [93] discussed the development of a practice for obtaining the vibration characteristics of wind turbine blades using a digital image correlation system installed on a UAV. The aim is to create a non-contact monitoring technique for wind turbines that is robust and does not interfere with their operation. The proposed health monitoring technique can be used by engineers for remote structural health monitoring of wind turbines during operation in both offshore and inland wind farms. Sanchez et al. [196] developed a new CM system for wind turbines based on deep acoustic analysis. The system includes an acoustic sensor embedded in a UAV, which allows for real-time monitoring of the turbines. They present a new approach for analyzing wind turbine noise patterns using frequency domain analysis and describe the results of laboratory tests and a real case study. Wang et al. [197] proposed a data-driven framework for automatically detecting wind turbine blade surface cracks using images from UAVs. The framework utilizes Haar-like features and an extended cascading classifier to locate and identify cracks, demonstrating its effectiveness through comparisons and computational studies. Icing is also a concern for FOWTs, especially for those located in Northern Europe and the Arctic, where there is a high risk of icing events. Gao et al. [198] used a DJI Mavic UAV equipped with a high-resolution camera to photograph iced turbine blades to stud icing on them.

Papers and projects about UAV applications in FOWT inspection in the past decade are summarized in Table 6 with their functions, technical characteristics, advantages, and disadvantages. Some technical details are not available because of protecting trade secrets. It can be concluded from the table, which presents a comprehensive analysis of various UAV inspection projects, that most of the current initiatives and implementations focus on non-contact monitoring techniques, primarily utilizing cameras, infrared cameras, or LiDAR technology. These cutting-edge UAV inspection projects have embraced advanced visual and sensing capabilities, allowing them to assess and inspect a wide range of objects and environments efficiently and accurately. In addition, another trend of these studies is that most of them focus on the inspection of blades, and there is still a lack of research on the detection of other FOWT components.

## 4. Challenges and Future of Prospects Results

It can be found by comparing UAVs with other methods and technologies commonly used for FOWT inspection that each approach has its own special and insurmountable drawbacks. Traditionally, manned helicopters have been used for FOWT inspections, while they are expensive and require trained pilots. Elevating platforms and ropes are labor-intensive, time-consuming, and carry safety risks for the technicians. Permanently fixed sensors installed on FOWTs provide continuous monitoring but lack the mobility and flexibility of UAVs. Meanwhile, developing reliable and durable permanent sensor solutions remains a challenge. The development of climbing robots that can handle the unique challenges posed by FOWTs, such as curved surfaces, variable geometries, and irregular access points, is still in its early stages. Achieving the necessary levels of dexterity, adaptability, and autonomous operation is a significant technical hurdle. As for ROVs, they are limited in their ability to inspect above-water structures. Finally, there are limitations in terms of flight time, payload capacity, and operability in adverse weather conditions for UAVs. FOWTs are often located in offshore areas with strong winds and challenging weather patterns, making it difficult for conventional UAVs to operate safely and reliably. Addressing these shortages requires continued research and development in the fields of robotics, sensor technology, and offshore engineering. Overcoming the challenges associated with manual inspections, permanent sensors, climbing robots, ROVs, and UAVs will contribute to safer and more efficient FOWT operations, ensuring the long-term viability and sustainability of offshore wind energy.

### 4.1. Challenges of Applying UAVs in FOWT Inspections

Advancements and the continual development of UAVs have been ongoing for decades, but the systems are still considered to be in the early stages, necessitating further validation of their reliability [39]. There are several challenges in the development of the UAV. Firstly, the restricted payload capacity imposes limitations on the size and weight of the devices that can be used. And the limited payload capacity restricts the size of battery packs, leading to shorter flight durations. Secondly, most of the research focuses on non-contact measurement, and there is a lack of research based on contact measurement. Thirdly, current embryonic legal frameworks that regulate UAVs present significant barriers to research and development. These challenges will drive engineers to design new robotic systems in future studies to provide a better platform for FOWT inspection missions. In addition to the existing UAV challenges, several new challenges, such as technical and standardization aspects [208], public safety and privacy [209], and mobility optimization [210] need further attention.

#### 4.1.1. Payload Capacity and Duration Time

Payload refers to the maximum weight a UAV can carry, impacting its endurance, communication, coverage, and operational altitude [211]. These can encompass a range of payloads, such as sensors for reconnaissance, video cameras for monitoring, and mobile phones aiding cellular communication. UAVs need to carry this equipment while maintaining stability and maneuverability, which can be challenging, particularly for smaller UAVs. Energy and communication problems also pose substantial hurdles in this scenario, further impeding the potential for conducting unmanned inspections of FOWTs [66]. As a UAV’s payload capacity increases, it accommodates various essential accessories. This expanded capacity facilitates diverse functionalities and applications. What is more, the lightweight nature of UAVs renders them susceptible to weather changes, particularly in adverse wind conditions. Therefore, UAVs operating at sea need greater weight and power to overcome the environmental impact.

Due to the limited total load capacity, there is a contradiction between carrying larger capacity batteries and more sensors or other devices. FOWTs are often situated far from the shore, making round trips for recharging or swapping batteries time-consuming and inefficient. Increasing the flight duration of UAVs without compromising on payload capacity is crucial to covering a larger area and inspecting multiple turbines in one flight session. Offshore harsh conditions like strong winds, saltwater exposure, and unpredictable weather can affect flight stability, reduce battery efficiency, and increase wear and tear on equipment, affecting both payload capacity and flight duration. In order to solve the problem of limited payload capacity and insufficient duration time, a feasible solution is to use a multi-robot system to charge or replace different payloads for UAVs at sea so as to improve the efficiency of a single flight of UAVs.

#### 4.1.2. Need of Contact Measurement

The application of UAVs in FOWTs inspection is still in its infancy, and the only research and commercial applications are mostly limited to the identification of blade surface faults through video and images captured by manual or computer vision analysis, which have great limitations compared with the traditional contact method for comprehensive inspection of wind turbines. And many researchers focus on the research of computer vision and artificial intelligence rather than the development and innovation of UAV itself. This is because it is relatively easy and mature to use the camera system mounted on the UAV platforms to collect video image signals, and the same platform can play a role in different tasks; even ordinary commercial consumer UAVs can do it. However, UAVs, as robots that can maintain their position and speed through aerodynamic forces, have great potential for physical interaction with their surroundings. In recent years, several projects have emerged to enable UAVs to interact with the physical environment by incorporating aerial manipulators into the UAVs [212,213,214,215,216,217,218,219,220,221,222]. Some can also be used for contact inspection of infrastructure, such as bridges [177,178,223,224]. Through the review of these projects, some possibilities and inspirations for applying UAVs to FOWTs inspection could be found.

Contact measurement requires the UAV’s moving components, like the propeller, to stay near the target being inspected. However, in a dynamic environment, this proximity increases the risk of collisions between the UAV and the target or surrounding obstacles, leading to potential accidents. The UAV’s unexpected failure during its mission can disrupt inspection operations, leading to a notable decrease in wind turbines’ electricity generation. Mahmood et al. [225] introduced a semiquantitative reliability analysis framework aimed at assessing the criticality of mission failures in inspection UAVs, addressing system and component levels.

#### 4.1.3. Policy and Law

Due to the rapid advancement in technology and the expanding operational capacities of UAV technology, its applications are constantly evolving and pose specific challenges for flight operators, end users, and aviation authorities, particularly surrounding privacy, data protection, and public safety within the aviation sector [226]. Current legal frameworks that regulate UAVs present significant barriers to research and development. Moreover, beyond the existence of legal frameworks, market influences, such as industry design norms and accessible information about UAVs as public assets, are anticipated to profoundly influence forthcoming advancements [56]. The wide array of stakeholders of the application of UAV includes governmental regulatory bodies, judicial entities, research institutes, public policy organizations, UAV manufacturers, technology developers, service providers, news agencies, insurance companies, non-profit organizations focused on public interests, privacy advocates, supporters, and critics of UAV use, both public and private institutions utilizing UAVs, as well as individual users [227]. Since the early 2000s, countries have been steadily implementing their own national legal structures for regulating UAVs. Despite sharing the overarching objective of reducing risks to both airspace users and people and property on the ground, the policies, regulations, and laws surrounding the inspection of infrastructure vary significantly from one country to another due to differences in governance structures, priorities, and resources. Ideally, standards should be established uniformly across governmental and private entities, spanning multiple countries [227].

Several review articles exploring UAV applications delve into the regulatory frameworks governing them [228,229,230]. These pieces outline national and international regulatory bodies while also providing brief overviews of risk-based approaches, classifications based on UAV safety levels, and ongoing endeavors by international organizations aimed at standardizing UAV legislation. Stocker et al. [56] outlined noticeable patterns derived from comparing regulations governing UAVs across 19 different nations. Presently, legal frameworks play a pivotal role in governing UAV usage. With the burgeoning UAV market, there is a probable shift toward market mechanisms gaining greater prominence. Moreover, emphasis is placed on information and education as facilitators for public acceptance and seamless integration of UAVs into society. However, the absence of political will remains a fundamental barrier to substantial changes. Despite regulations existing in approximately one-third of countries, deficiencies in enforcement and implementation capabilities create noticeable gaps and limitations [56].

The EU regulation replaced all national rules in 2020, aiming to establish a unified UAV market in Europe that prioritizes safety. The reformed structure introduces three operation categories—open, specific, and certified—based on risk levels. Low-risk operations (open category) will not necessitate prior authorization but will have strict operational limitations. For medium-risk operations (specific category), operators must seek authorization from the national aviation authority through a standardized risk assessment or specific scenario approval. High-risk operations (certified category) will adhere to traditional aviation rules. Open category operations are limited to visual line of sight (VLOS) operations below 120 m altitude. UAVs in the open category will display a class identification label to demonstrate compliance. Each class of drone faces additional operational restrictions, especially concerning the distance maintained between the drone and non-involved persons (Table 7).

Significant risks of UAVs include malfunctions, mid-air collisions, and resulting harm to individuals and property below. Regulations governing UAVs primarily center on three key areas: controlling their airspace use, setting operational boundaries, and managing administrative processes involving flight permissions, pilot licensing, and data collection authorization. Shared requirements like mandatory platform registration, necessary insurance, and standardized pilot licensing demonstrate a growing trend in national UAV regulation maturity. One additional hurdle faced by the implementation of UAVs is the regulation concerning operators. Commercial operation of UAVs, whether remote-controlled or autonomous, mandates pilots to obtain a suitable license. Meeting the certification requirements necessitates expensive and comprehensive training programs as well as accumulating a significant number of flight hours. Nonetheless, aviation authorities continuously revise regulations to align with societal needs, and an increase in pilot licensing is anticipated alongside these advancements [39].

### 4.2. Solution 1: Multi-Robot System

Although UAVs have flexible movement ability, their limited endurance is their weakness, and compared with fixed-wing UAVs, the airspeed of multi-rotor UAVs is not high. Currently, the global averages of distance to shore and water depth are 18.8 km and 14.6 m, respectively. These dimensions for the power plants installed in Europe have an average of 23.3 km and 17.4 m [1]. It is logistically easier to inspect onshore wind turbines and fixed offshore wind turbines with UAVs, while FOWTs are far from the coast, and it is difficult to meet the requirements of flying UAVs from the coast to FOWTs with a one-time charge.

The rapid advancement of diverse autonomous mobile robotics has led to an increased significance of cross-domain heterogeneous robot systems. These systems are increasingly crucial in comparison to human operators, particularly for performing complex tasks in monotonous, dangerous, and inaccessible environments [231]. By combining UAVs with other robot platforms and leveraging the respective advantages of each to build a multi-robot system, researchers can capitalize on the endurance benefits and capabilities unique to each platform. This synergistic collaboration allows for a more comprehensive and robust system that surpasses the limitations of individual technologies. For instance, the UAV’s expansive coverage and aerial maneuverability can be harnessed in conjunction with the endurance advantages of ground-based or marine robots. Conversely, the integration of UAV transport platforms with other robots yields reciprocal benefits. By utilizing UAVs as transport mechanisms, other robots can extend their range of activities beyond their inherent mobility limitations. This augmentation in operational scope not only enhances their overall efficiency but also facilitates automation and the completion of tasks that were previously unattainable.

**Figure 13:**
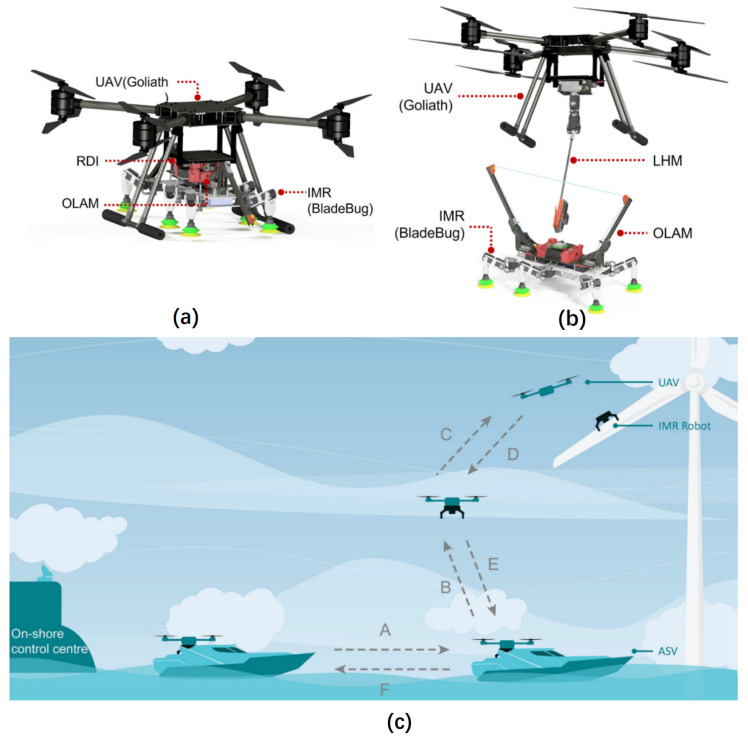
Multi-robot system for FOWT inspection, adapted with permission from Ref. [98], copyright ©2022 Wiley Periodicals LLC. (**a**) The UAS for deployment (Goliath + RDI) and the IMR robot system (OLAM + BladeBug); (**b**) the UAS for retrieval (Goliath + LHM) and the IMR robot system; (**c**) illustration of the maintenance and repair in extreme environment multi-robot system for offshore wind farm O&M.

Bernardini et al. [95] presented a proposal for an advanced, collaborative, unmanned, multi-robot system intended for the autonomous inspection, maintenance, and repair of offshore physical assets, integrating multiple robotic platforms into a cohesive, coordinated system cooperating with human operators located onshore. The platform (see Figure 13) includes an ASV, which is equipped with a team of robotic UAVs containing a fleet of modular UAVs with reconfigurability onboard the ASV and climbing robots deployed onto stationary wind turbine blades to carry out subsurface NDT inspections. In this project, the mothership navigates to the offshore assets and conducts continuous inspection, maintenance, and repair operations for extended periods, even under adverse weather conditions.

In summary, using a multi-robot system for floating offshore wind turbine inspection offers advantages such as improved efficiency, increased coverage, redundancy, specialized capabilities, flexibility, adaptability, and long-term cost-effectiveness. These benefits make multi-robot systems a promising solution for optimizing the inspection process and ensuring the reliable and safe operation of floating offshore wind turbines.

### 4.3. Solution 2: Contact Inspection Method

The use of UAV systems equipped with remote sensing payloads has greatly reduced monitoring efforts. However, most existing inspection methods primarily identify surface damages [184]. To detect internal structural issues, NDT methods rely on sensors such as ultrasounds, accelerometers, and resistivimeters, requiring direct contact with the structure. Vibration analysis is pivotal in CM systems for identifying potential issues within machinery. It stems from misalignments and surface wear in rotating parts, generating noticeable vibrations. These vibrations directly reflect machine dynamics, making vibration analysis an invaluable, non-intrusive tool for diagnosing faults [82].

Contact measurement is difficult for UAVs, but the advantages brought by physical contact cannot be replaced by other inspection methods, so some structures could be added to UAVs to overcome this insufficient. However, due to aerodynamic turbulence and concerns regarding potential collision damage, it is imperative for UAVs to maintain a limited standoff distance from the asset [178]. Furthermore, to achieve a longer flight time, the UAV load should be as light as possible. In contrast, the imperative for achieving lightweight designs has consequently rendered UAVs more vulnerable to perturbations during their operational missions. Therefore, it is necessary to study how to make the UAV establish physical contact with the wind turbine while remaining lightweight and safe. The UAV can be in two situations when it contacts the inspected object: the landing and the in-air situation.

Non-contact methods are limited to the detection of visual or infrared faults and surface-exposed defects, which imposes constraints on certain inspection tasks. When significant damage is detected, a comprehensive examination must ensue, requiring experienced inspectors to have hands-on access to the FOWT section [177]. Therefore, when dealing with structural health conditions like subsurface corrosion beneath the outer facade, the use of contact measurement technologies becomes necessary. Contact methods [177,178,223] provide the advantage of direct sensory feedback, enabling inspectors to perceive characteristics such as texture, hardness, or vibrations. However, in the context of UAV inspections, maintaining a significant standoff distance is typically essential to prevent collisions. This poses a considerable challenge for UAV pilots when attempting to conduct close-range and contact examinations due to obstructed views of pilots [178].

#### 4.3.1. Landing Contact

**Figure 14:**
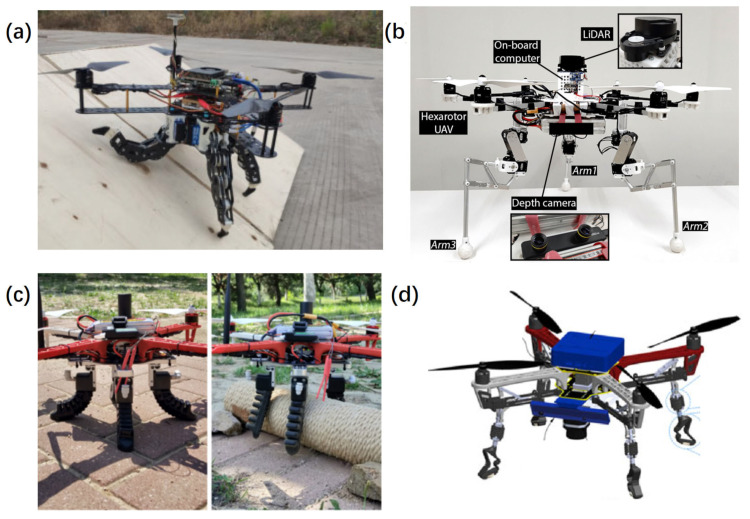
UAVs with deformable landing gear. (**a**) A novel multi-finger robot landing gear, adopted with permission from Ref. [232], copyright ©2021 Elsevier B.V.; (**b**) UAV with parallel link three-manipulator landing system, adapted with permission from Ref. [233], copyright ©2021 MDPI; (**c**) UAV successfully landed on convex surfaces with the support of the proposed soft landing gear, adopted with permission from Ref. [234], copyright ©2021 MDPI; (**d**) Bioinspired design of a landing system with soft shock absorbers, adopted with permission from Ref. [235], copyright ©2018 Wiley Periodicals Inc.

In the process of using UAVs to inspect FOWTs, the conventional contact method requires the UAV to hover for extended periods, utilizing its propellers to apply pressure onto sensors and the target surface. While this method is relatively straightforward to implement, it significantly reduces the UAV’s flight endurance, and the instability during hovering can introduce interference and reduce the quality of collected data. Therefore, a more effective solution involves landing the UAV directly on the target platform for inspection. By employing a landing-based approach, the UAVs can establish a stable connection with the FOWTs, mitigating the adverse effects of prolonged hovering. This not only enhances the UAV’s endurance but also allows for a more reliable and prolonged data collection process. Landing a UAV on a moving and uneven target in a difficult and dynamic environment is a challenging task. There have been some mature research projects in this area, much of them are based on computer vision. There are also projects on landing gear designed to land on dynamic or uneven surfaces, as shown in Figure 14.

The existing UAV landing gear program can be divided into active and passive. The actively adapted landing gear is composed of mechatronic systems, which change the shape of the mechanical structure through the motor or the steering gear to maintain the level and stability of the UAVs landing in different environments, as shown in Figure 14a,b. These systems work as robot arms. Therefore, the study of actively adapted landing gear is often accompanied by aerial grasping. In the process of inspecting FOWTs using UAVs, the development of deformable or flexible landing gear is necessary to achieve landing on moving targets. FOWTs are subject to wave and wind-induced movements. Deformable or flexible landing gear allows the UAVs to adjust their landing approach dynamically, compensating for the platform’s motion and ensuring a safer and more stable landing, and reducing the risk of accidents and collisions during landing. Zhang et al. [235] explored bioinspired solutions in adaptive landing gear morphologies of small aerial robots to achieve dynamic landing on varied surface structures as well as moving targets, as shown in Figure 14c,d.

However, there are some significant drawbacks to post-landing testing. Firstly, with regard to safety, it is prone to accidents during the landing and takeoff process. Most aircraft accidents occur during takeoff and landing. During takeoff and landing, UAVs are subject to turbulence and require more complex manual or automated controls than in the air. While specially designed landing gear can improve safety, unnecessary takeoffs and landings themselves pose risks that cannot be ignored. Secondly, UAVs are difficult to land on sloping, smooth surfaces, especially vertical ones, because it is difficult for UAVs to grasp the surface and take off in a non-horizontal attitude.

Although UAVs possess advanced sensors and control algorithms, their performance is significantly influenced by variations in wind speed and direction, which, in turn, affect the motion of the UAVs. Any unanticipated malfunctions of the UAV system throughout the landing and taking off have the potential to disrupt inspection operations, consequently resulting in a substantial decrease in the electricity production facilitated by wind turbines. Due to these shortcomings, landing a UAV on a wind turbine is a flawed solution, and researchers need to explore contact methods that are more adaptable to complex environments.

#### 4.3.2. Aerial Manipulator

**Figure 15:**
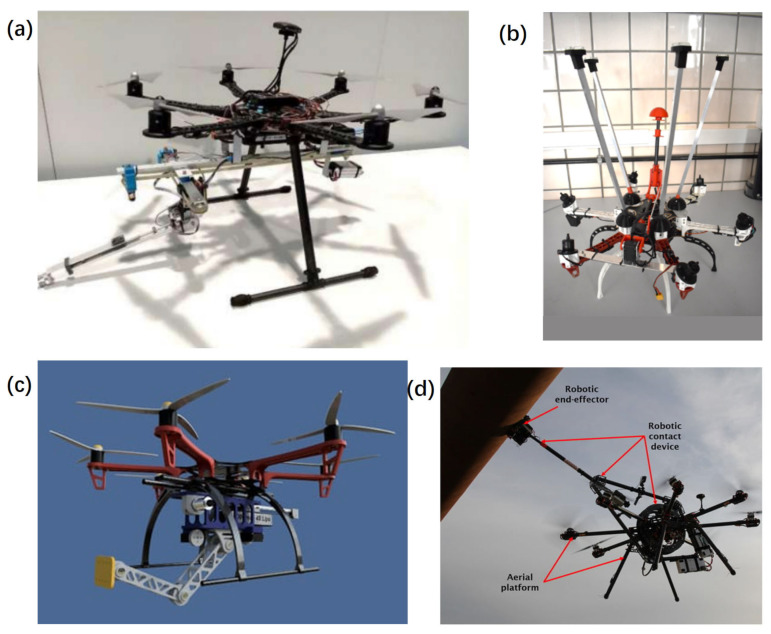
Existing aerial manipulators. (**a**) Cartesian aerial manipulator, adapted with permission from Ref. [221], copyright ©2021 MDPI; (**b**) fully actuated aerial manipulator for infrastructure contact inspection, adapted with permission from Ref. [177], copyright ©2020 MDPI; (**c**) a lightweight cable aerial manipulator for construction inspection purposes, adapted with permission from Ref. [238], copyright © 2022, MDPI; (**d**) the AeroX robot in physical inspection of a pipe during a validation experiment, adapted with permission from Ref. [239], copyright © 2019 MDPI.

In recent years, the emergence of robotic aerial manipulators has significantly broadened the potential applications of UAVs [177]. Aerial manipulators [212,236], referring to aerial robots equipped with arms or mechanical devices, possess the capability to engage in physical interactions with the environment. This enables them to carry out a diverse range of tasks, including object manipulation [215], operating devices [216,217], aerial 3D printing [213,218], and conducting measurements through sensors in contact with surfaces [177,178,223]. Such capabilities also allow UAVs to not only be limited to non-contact inspection of targets but also to engage in some physical contact-based interaction. While there are currently no UAV aerial manipulator projects specifically for contact inspection of wind turbines, there are already projects for other infrastructure such as bridges [237], buildings, etc., which is shown in Figure 15.

The research on contact inspection methods specifically applied to FOWTs is very limited, but there are many studies for other infrastructures, such as bridges and tunnels. Sanchez-Cuevas et al. [177] presented the design, modeling, and control of a fully actuated UAV for infrastructure contact inspection as well as its localization system, which could be used in the inspection of FOWTs. The UAV is equipped with a 3DoF lightweight arm, which can measure the contact force to regulate the force applied to the sensor on the structure. And the Horizon 2020 framework program [240,241,242] includes a number of projects that apply contact methods to infrastructure inspection using aerial manipulators. All these inspection techniques can be applied to FOWTs after conversion. González-deSantos et al. [237] introduced a payload created for semi-autonomous collision-mitigation inspections on UAVs. The system operates independently without relying on external positioning aids like motion capture systems or GPS.

The existing aerial manipulator projects mostly involve shock absorption or simple action by the robot arms and then the pressure exerted by the UAV motors’ own power on the target so that the sensor is pressed against the inspected object. Researchers need to explore aerial manipulators that can overcome the shortcomings of these projects:

1. Due to asymmetry, it is impossible to maintain the center of gravity of the UAV in the geometric center, resulting in wasted power, increased control difficulty, and increased risk.
2. The connection between the UAV and the detected object is rigid, but the UAV needs to constantly adjust its attitude to move and maintain stability. And the movement of motors, propellers, and other components creates constant noise, which makes the contact unstable and affects the work of the sensor.
3. The weight of these air manipulators is very large, and the movement inertia is also very large, which will cause a large disturbance to the UAV during the inspection and reduce its endurance.

#### 4.3.3. Parallel Aerial Manipulator

The combination of parallel manipulator and UAV has the potential to overcome the above defects. By installing a delta parallel robot on the UAV to compensate for the motion of the UAV, Professor Zhang Ketao realized accurate dynamic control of the end effect to realize in-air 3D printing [218]. The excellent characteristics of parallel robots make it possible to control the end effect accurately and quickly.

**Figure 16:**
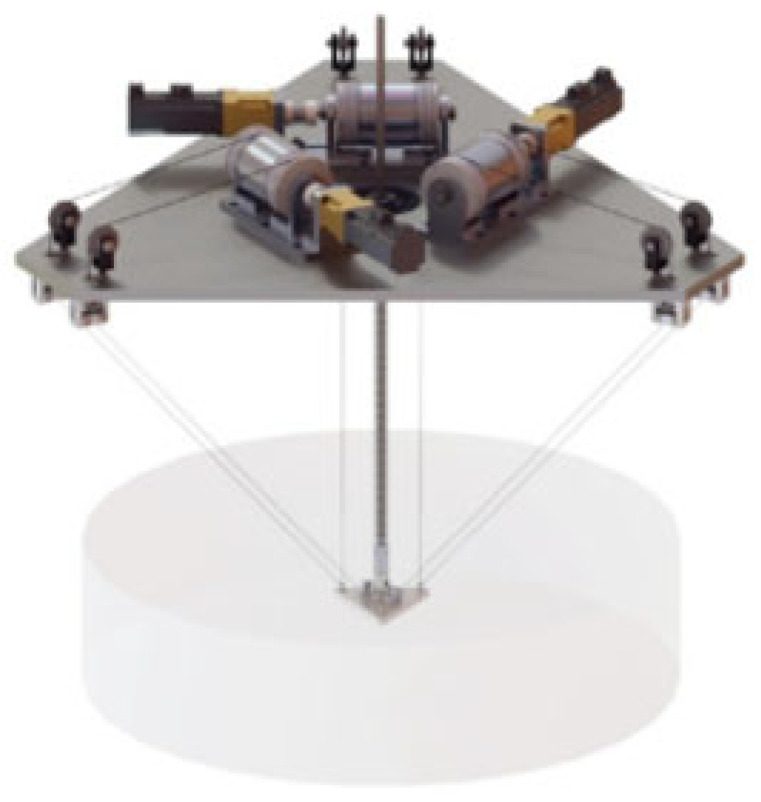
High-speed pick-and-place cable-driven parallel robot with the potential to be integrated with UAVs, adapted with permission from Ref. [249], copyright ©Zhaokun ZHANG et al.

In addition to the delta robot, there are other parallel robotic arms that have the potential to be combined with UAVs. Cable robots represent arguably the most lightweight configuration for a manipulative system, which is particularly valuable for UAVs [243]. Dr. Zhaokun Zhang has designed a cable-driven parallel robot with three sets of cables and a central spring rod that can achieve higher speeds, more precise control, lighter weight, and lower energy consumption than delta parallel robots [244,245,246,247,248,249], as shown in Figure 16. The innovation of the design is the use of retractable spring rods to keep the cable taut rather than relying solely on gravity, allowing for rapid robot movement. This design has great potential to be applied to my project.

At present, there is no research on the combination of cable-driven parallel robots and UAVs. Since the force can only be transmitted one way in the cable, and the cable must be in a tensioning state, the cable-driven parallel robot can realize the physical contact between the UAV and the inspected target while eliminating unnecessary force transmission. The use of cables enables the robot to have a larger workspace, lighter mass, higher operation speed, and larger payload-to-weight ratio [250].

#### 4.3.4. Rotor-Distributed Manipulator

**Figure 17:**
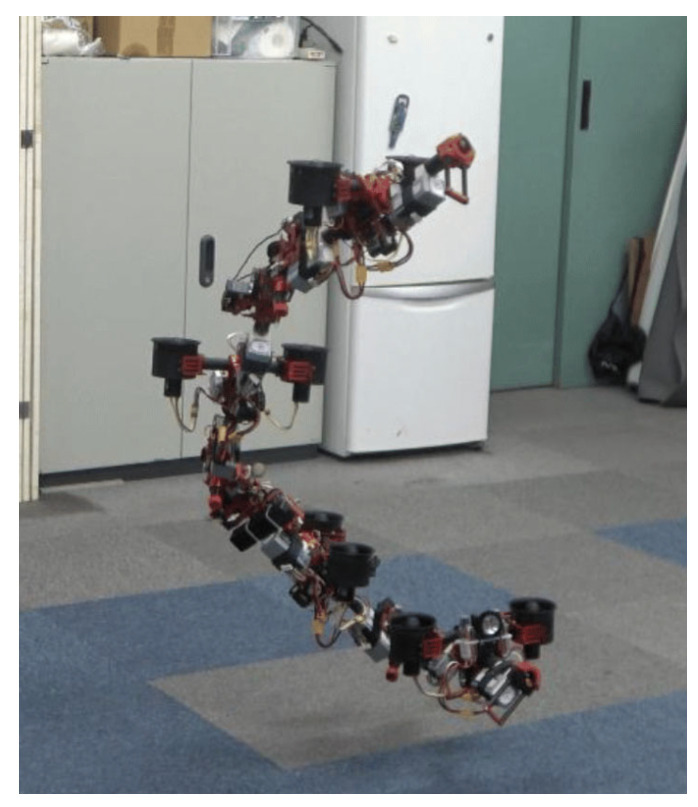
Modular snake-like aerial robots with four links and four thrust vectoring apparatus, adapted with permission from Ref. [267], copyright ©2023 MDPI.

Traditional aerial manipulators encounter three primary challenges. First, their end effector stability in the air proves inadequate for handling more complex tasks. Second, due to limitations in payload capacity, these manipulators face strict constraints on arm joint torque, resulting in the generation of a relatively small wrench at the end effector. Lastly, the motion of the end effector gets disrupted by the concentrated rotors used in conventional aerial manipulators, specifically multi-rotors with arms [251]. In addressing these challenges, several innovative approaches have emerged. The perching methodology encompasses adhesive [252,253,254,255,256,257], gripper [258,259,260], magnetic [261,262], and rotor suction force [263] techniques, each tailored for specific surfaces but constrained by surface type limitations. The second method, the rotor-distributed manipulator (RDM), strategically places rotors along arm links to overcome limitations in feasible wrenches, enabling greater maneuverability and versatility in aerial robotics [264,265,266]. Zhao et al. [267] introduced a rotor-distributed aerial robot, shown in Figure 17, featuring links interconnected by two servo motors. However, while larger-scale rotor-distributed robots exhibit increased wrench potential, their size and complexity pose practical deployment challenges. Lastly, the root-perching technique focuses on maximizing reachability using a minimal configuration RDM, emphasizing perching stability considering contact conditions like static friction and zero moment point. To increase the reachability at the end effector, the manipulator only uses a part of its body for perching, such as the root unit. Nishio et al. [251] designed a minimal RDM with root-perching abilities. Additionally, they presented a control technique aimed at minimizing rotor thrust during perching while accounting for foot conditions and a motion planner, and they conducted reachability assessments for RDMs to gauge their effectiveness.

The ability of the RDM robot root to absorb on the surface can improve the stability of the UAV so as to overcome the adverse factors of instability in the marine environment, and compared with traditional UAV, RDM can apply greater torque, meaning that it can carry heavier sensors for inspection, and can apply greater loads to the target, and can also perform basic actions such as drilling and painting. Potential for maintenance of FOWTs. Due to its deformable and reconfigurable characteristics, RDM can enter the narrow and complex environment that traditional UAVs cannot enter and has a better ability to perform tasks.

### 4.4. Prospects of UAV-Based Inspection

By comparing different inspection methods, it can be concluded that UAVs have better performance and potential compared to manual, permanent sensors and other robotic inspection methods. However, due to disadvantages such as limited range and easy interference during flight, the future of UAVs for FOWT inspection will focus on cooperation with other robotic platforms and combination with aerial manipulators. Table 8 compares the properties of cable-driven parallel manipulators, delta parallel manipulators, and series manipulators in terms of center of gravity position, working space, material, and weight. The cable-driven parallel manipulator has the best performance. Therefore, the combination of cable-driven parallel manipulators with UAVs is considered a potentially valuable research direction in the future.

What is more, UAVs always tend to focus on exterior and NDT inspections, but their use for interior inspections is still limited. Kulsinskas et al. [199] discussed the viability of using UAVs for interior wind turbine blade inspections, which are essential for extending the lifetime and improving the efficiency of wind turbines. The challenges of performing interior inspections include confined spaces, lack of illumination, and potentially harmful internal structural components, as well as issues. Exterior inspections and NDTs predominantly yield essential information regarding potential defects in FOWTs. However, it is important to acknowledge that defects may also manifest exclusively within the interior, necessitating thorough inspections conducted from within the turbine itself. Therefore, the use of UAVs for atypical site inspection is also one of the potential research directions in the future.

## 5. Conclusions

This research investigation is the first to provide a holistic overview of the status of the inspection of FOWTs using multi-rotor UAVs. It further delivers insights into the past, present, and future development of UAVs as well as their application in other industries. Based on a research synthesis that includes a thorough literature review and comparative analysis of different inspection methods for FOWTs, similarities and contrasting elements in various methods and their advantages and disadvantages are explored. Then, the paper summarizes the advantages and prospects of using UAVs in this field. The main findings of this review are:

1. Using UAVs instead of conventional manual inspection methods offers significant cost reduction for FOWT inspection, minimizes downtime, and ultimately contributes to lowering the unit energy price, fostering sustainable societal development. Additionally, it can play a pivotal role in mitigating H&S hazards;
2. Compared with other robotic systems and permanent sensor CM systems, UAVs are the most promising competitors for future development due to their low cost, fast deployment, high mobility, and strong reusability;
3. The current applications of UAVs in FOWT inspection predominantly utilize computer vision, infrared, and LiDAR technologies, focusing on non-contact measurements. The primary research efforts have centered around visual algorithms, resulting in significant innovations in this area. However, there is limited exploration of other sensing techniques, such as vibration analysis and ultrasonic, as well as emphasis on innovations in UAV mechanical design, embedded systems, and multi-sensor fusion;
4. UAV contact-based measurement methods are in their early stages, with some research already conducted, but there remains ample room for further advancements. The combination of UAV and parallel aerial manipulator is a promising research direction in the future;
5. Since the inherent defects of a single robotic platform, including UAV, are difficult to overcome, the use of multi-robot systems for UAV inspection of FOWTs is a promising avenue that remains largely unexplored.

One noteworthy consideration for researchers is that UAV methods are designed not to replace, compete with, or eliminate existing inspection methods but rather to compensate for some of their inherent shortcomings. Functioning as an enabling technology, the primary role of UAVs is to augment and facilitate the utilization of other inspection technologies through their consistently updated advanced technology and robust motion capabilities. Clarifying this objective will guide researchers in the right direction for scientific inquiry. The development of alternative inspection methods not only avoids competition with UAVs but also allows for collaboration, leveraging multi-robot systems and aerial manipulators to collectively advance industry development. Addressing these research gaps could result in more efficient, reliable, and comprehensive inspections of FOWTs, ultimately contributing to the sustainable development and maintenance of offshore wind energy systems. This, in turn, aids in reducing the cost per unit of energy and contributes to a more environmentally friendly planet.

## Figures and Tables

**Table 2:** The monitoring and analysis techniques of different components. (The “✓” indicates that the technique has been applied to the inspection of the component.).

Techniques	Nacelle	Tower	Blade	Bearings	Shaft	Gearbox	Generator
Vibration analysis	✓	✓	✓	✓	✓	✓	✓
Torsional vibration					✓	✓	
Acoustics emission		✓	✓	✓	✓	✓	
Oil analysis				✓		✓	✓
Strain measurement		✓	✓				
Optical fiber monitoring			✓				
Electrical effects				✓			✓
Temperature	✓			✓		✓	✓
Ultrasonic testing techniques		✓	✓				
Thermography	✓		✓	✓	✓	✓	✓
Visual inspection	✓	✓	✓				
Radiographic inspection		✓	✓				
Generator power output							✓

**Table 3:** Risk matrix.

			Severity				
			1	2	3	4	5
			Very Minor/No Injuries	Moderate Injuries	Lost Tim Injuries	Serious Injuries /Permanent Disabilities	Fatalities
Likelihood	5	Almost Certain	Low Medium (5)	Medium (10)	High (15)	Very High (20)	Very High (25)
	4	Very Likely	Low Medium (4)	Medium (8)	Medium (12)	High (16)	Very High (20)
	3	Likely	Low (3)	Low Medium (6)	Medium (9)	Medium (12)	High (15)
	2	Unlikely	Low (2)	Low Medium (4)	Low Medium (6)	Medium (8)	Medium (10)
	1	Very Unlikely	Low (1)	Low (2)	Low (3)	Low Medium (4)	Low Medium (5)

**Table 4:** Summary of the most serious hazards of each method.

Method	Hazards Identified and Potential Harm It Could Cause	Likelihood	Severity	Risk Rating
Manual	Fell from a height Drowning Get an electric shock	3 Likely	5 Fatalities	15 High
Permanent Sensors	Short circuit caused by line aging	1 Very unlikely	1 Slight injury, no treatment required; no time lost	1 Low
Climbing Robot	The crew on the support vessel fell overboard during the launch	3 Likely	3 Lost time injury	9 Medium
ROVs	The crew on the support vessel fell overboard during the launch	3 Likely	3 Lost time injury	9 Medium
UAVs	UAV crashes out of control	3 Likely	1 Slight injury, no treatment required; no time lost	3 Low

**Table 5:** Summary of the advantages and disadvantages of the different methods for the inspection of FOWTs.

Methods	Sensors	Advantages	Limitations
Manual inspection	Visual	Direct human intervention High mission diversity.	Time-consuming, costly H&S risk Limited access to certain areas, subjective assessment.
Permanent sensor	Vibration sensor, temperature sensor, speed sensor, strain sensor	Continuous monitoring, real-time data collection, less human intervention required, early detection of anomalies.	Limited to specific sensors and sites, may not capture comprehensive data, inability to perform physical repairs, require additional inspection methods for detailed assessment.
Climbing robot	Visual	Can access vertical surfaces, perform close-range inspections, suitable for complex structures, collect visual and sensor data.	Limited to vertical surfaces, may require complex deployment mechanisms, slower inspection process, higher cost compared to UAVs.
ROV	Visual, sonar	Can inspect underwater structures, perform detailed inspections, collect visual and sensor data, suitable for subsea components.	Limited to underwater inspections, require complex deployment and operation, higher cost, restricted to specific areas, challenging in harsh weather conditions.
UAV	Visual, LiDAR, GPS	Versatile and flexible, can perform visual and thermal inspections, capture high-resolution imagery, cover large areas quickly, collect comprehensive data, cost-effective.	Limited flight endurance, weather-dependent, may require skilled operators, challenges in confined spaces or high winds, manually restricted to line-of-sight operations.

**Table 6:** Summary of the papers and projects of UAV inspection of FOWTs. (For certain fundamental applications, there are not obvious advantages and disadvantages that can be identified.).

Components	Result	Technique	Sensors & Hardware	Evaluation	Source
			Papers		
Blade	Defect Identification	Obtaining the vibration characteristics using a digital image correlation (DIC) system installed on a UAV	Cameras	Pros: Non-contact vibration monitoring Cons: Crating pattern on utility scale wind turbine can be a challenge	Khadka et al. [93]
Blade	3D Modeling Defect Identification	High-resolution blade images and a 3D model of the wind-turbine structure	LiDAR	Pros: No additional pre-flight setup required Cons: requires some manual operation	Car et al. [94]
Blade	Navigation	Autonomous machine vision navigation	Camera	Pros: ability to capture the main features with low computational demand Cons: exclusive focus on the initial stage of the UAVs’ operation	Stokkeland et al. [85]
Blade	Defect Identification	Detecting wind turbine blade surface cracks using images from UAVs	Camera	Pros: The inspection process could be completed rapidly Cons: Not Available	Wang et al. [197]
Nacelle	Defect Identification	Ultrasonic Inspection by a ultrasonic sensor	ultrasound probe Camera LiDAR	Pros: Contact method Cons: Only laboratory tests were carried out.	Zhang et al. [178]
			Projects		
Tower Blade	Navigation 3D Modeling Defect Identification	Geometry-based path planner for coverage of complex structures. Flexible localization using UWB fused inertial estimation scheme visual 3D model building	Camera LiDAR UWB node IMU	Pros: reach high level of autonomy on a large scale Cons: 1. sensitive to the existing weather conditions 2. Wi-Fi is not a reliable communication 3. UWB anchors need to be kept permanently	Kanellakis et al. [172]
Blade	Defect Identification	Infrared Thermography	Infrared Camera	Pros: Being able to detect defects below the surface Cons: Low degree of automation	Galleguillos et al. [175]
Blade	Navigation Defect Identification	Computer Vision	Camera	Pros: Innovative the interior wind turbine blade inspections Cons: sensitive to the existing weather conditions	Kulsinskas et al. [199]
Blade Tower	Defect Identification	Close Visual thermal inspections	Camera LiDAR	Not Available	TERRADRONE [200]
Blade Tower	Defect Identification	Computer Vision, Autonomous navigation	Camera	Not Available	3DX [201]
Blade Tower	Defect Identification	Computer Vision	Camera	Not Available	Blade Edge [202]
Blade Tower	3D Modeling Defect Identification	Computer Vision Autonomous navigation	Camera LiDAR	Pros: The UAV can do the inspection automatically and save time. Cons: The project is not complete, and the hardware needs to adopt to maritime environment.	Alerion [184,185,186]
Blade	Defect Identification	Infrared Thermography	Infrared Camera	Pros: A unique and proven technology with high-resolution up to 3 mm Cons: Manually operated	ABJ [203]
Blade	3D Modeling Defect Identification	Computer Vision Autonomous navigation	Camera LiDAR	Pros: The global leader in turbine blade inspections with more than 200,000 successful inspections Cons: Not Available	SkySpecs [204]
Blade	Defect Identification	Computer Vision Infrared Thermography	Infrared Camera	Pros: Ability to work in harsh environments Cons: Not Available	Aerial Tronics [205]
Blade	Defect Identification	Computer Vision	Camera	Not Available	Clobotic [206]
Blade	Defect Identification	Computer Vision	Camera LiDAR	Not Available	Aero-Enterprise [207]

**Table 7:** Summary of the operations authorized in the open category for each class of drones as defined currently by EASA.

UAS		Operator/Pilot			Operator/Pilot	
Class	Weight	Subcategory	Operational Restrictions	Distance from People	Operator Registration Required	Remote Pilot Competence
Privately built	<250 g	A1	Operate in visual line of sight below 120 m altitude Fly away from airports Respect specific rules defined by the zone in which you operate	You can fly over uninvolved people (not over crowds)	No	Read owner manual
C0						
C1	<900 g				Yes	Read owner manual Perform online training Pass online test
C2	<4 kg	A2		You can fly at a safe distance from uninvolved people		Read owner manual Perform online training Pass online test Pass a theoretical test in a center recognized by the aviation authority
C3	<25 kg	A3		Fly in an area where it is reasonably expected that no uninvolved people will be endangered. Keep a safe distance from urban areas		Read owner manual Perform online training Pass an online test
C4 (model aircraft)						
Privately built						

**Table 8:** Comparison of cable-driven parallel manipulator, delta parallel manipulator, and series manipulator.

	Cable-Driven Parallel	Delta Parallel	Series
Center of gravity	Center of horizontal geometry	Center of horizontal geometry	Not in the center of horizontal geometry
Workspace	Large	Large	Small
Materials	Plastic or carbon fiber	Plastic or carbon fiber	Metal
Weight	Low	Low	High
Moment of inertia	Low	Low	High
Control accuracy	High (no error accumulation)	High (no error accumulation)	Low (error accumulation)
Connection form	Rigid connection and flexible connection	Rigid connection	Rigid connection

## Abbreviations

The following abbreviations are used in this manuscript:

AGV	Automated guided vehicle
AI	Artificial intelligence
ASV	Autonomous surface vessel
AUV	Autonomous underwater vessel
CAGR	Compound annual growth rate
CM	Condition monitoring
FOWT	Floating offshore wind turbine
GNSS	Global navigation satellite system
GPS	Global positioning system
H&S	Health and safety
IBVS	Image-based visual servoing
IMU	Inertial measurement unit
IoT	Internet of Things
IRT	Infrared thermography
LCOE	Levelized cost of energy
LiDAR	Light detection and ranging
MEMS	Micro-electro-mechanical system
NDT	Non-destructive testing
O&M	Operation and maintenance
RDM	Rotor-distributed manipulator
RF	Radio frequency
ROV	Remotely operated vehicle
SLAM	Simultaneous localization and mapping
UAS	Unmanned aerial system
UAV	Unmanned aerial vehicle
UMS	Unmanned systems
USV	Unmanned surface vehicle
UWB	Ultra-wideband
VLOS	Visual line of sight
